# TEAD1‐Mediated Trans‐Differentiation of Vascular Smooth Muscle Cells into Fibroblast‐Like Cells Contributes to the Stabilization and Repair of Disrupted Atherosclerotic Plaques

**DOI:** 10.1002/advs.202407408

**Published:** 2024-12-12

**Authors:** Ming Zhai, Zhijun Lei, Yefei Shi, Jiayun Shi, Yanxi Zeng, Shiyu Gong, Weixia Jian, Jianhui Zhuang, Qing Yu, Mark W. Feinberg, Wenhui Peng

**Affiliations:** ^1^ Department of Cardiology School of Medicine Shanghai Tenth People's Hospital Tongji University Shanghai 200072 China; ^2^ Department of Endocrinology School of Medicine Xinhua Hospital Shanghai Jiaotong University Shanghai 200092 China; ^3^ Cardiovascular Division Department of Medicine Brigham and Women's Hospital Harvard Medical School Boston MA 02115 USA

**Keywords:** ACS, atherosclerotic plaque, plaque rupture, TEAD1, thrombosis

## Abstract

Atherosclerotic plaque rupture mainly contributes to acute coronary syndrome (ACS). Insufficient repair of these plaques leads to thrombosis and subsequent ACS. Central to this process is the modulation of vascular smooth muscle cells (VSMCs) phenotypes, emphasizing their pivotal role in atherosclerotic plaque stability and healing post‐disruption. Here, an expansion of FSP1^+^ cells in a tandem stenosis (TS) model of atherosclerotic mice is unveiled, predominantly originating from VSMCs through single‐cell RNA sequencing (scRNA‐seq) analyses and VSMC lineage tracing studies. Further investigation identified TEA domain transcription factor 1 (TEAD1) as the key transcription factor driving the trans‐differentiation of VSMCs into fibroblast‐like cells. In vivo experiments using a TS model of plaque rupture demonstrated that TEAD1 played a crucial role in promoting plaque stability and healing post‐rupture through pharmacological or TEAD1‐AAV treatments. Mechanistically, it is found that TEAD1 promoted the expression of fibroblast markers through the Wnt4/β‐Catenin pathway, facilitating the trans‐differentiation. Thus, this study illustrated that TEAD1 played a critical role in promoting the trans‐differentiation of VSMCs into fibroblast‐like cells and subsequent extracellular matrix production through the Wnt4/β‐Catenin pathway. Consequently, this process enhanced the healing mechanisms following plaque rupture, elucidating potential therapeutic avenues for managing atherosclerotic instability.

## Graphical abstract

This study investigates the crucial role of VSMC trans‐differentiation into fibroblast‐like cells in stabilizing atherosclerotic plaques after rupture. It identifies TEAD1 as a key regulator of this process through the Wnt4/β‐Catenin signaling pathway, highlighting novel therapeutic targets for enhancing plaque healing and stability amidst inflammatory challenges. Discover how these insights can transform atherosclerosis treatment.

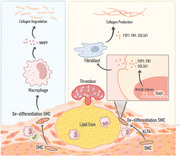

## 1 Introduction

Vulnerable plaques, with their hallmark thin fibrous caps and necrotic lipid cores, predispose individuals to thrombosis and acute coronary syndrome (ACS) when they rupture or erode.^[^ ^1^, ^2^, ^3^ ^]^ Nonetheless, advancements in intravascular imaging technologies, such as intravascular ultrasound (IVUS) and optical coherence tomography (OCT), have revealed that a reparative process often coincides with the rupture of these plaques, particularly when the associated thrombi are small.^[^ ^4^, ^5^, ^6^, ^7^, ^8^, ^9^ ^]^ This repair response may help counteract the destabilization of the plaques, supported by clinical evidence that some patients with vulnerable plaques exhibit resistance to developing ACS.^[^ ^10^, ^11^ ^]^ However, the balance is precarious: a shift toward larger thrombotic events and significant myocardial infarction can abruptly disrupt this reparative process, culminating in the onset of ACS.^[^ ^12^ ^]^ Therefore, timely and effective repair of ruptured plaques is essential to reduce the risk of ACS; yet the underlying mechanisms of these reparative processes are not well elucidated.

In healthy blood vessels, vascular smooth muscle cells (VSMCs) maintain a stable, contractile state that is essential for normal vessel function. However, in response to injury, aging, or conditions such as atherosclerosis, VSMCs lose their contractile characteristics and undergo de‐differentiation. During this process, Shankman et al. found that Kruppel‐like factor 4 (KLF4) acted as a molecular fate switch, promoting the de‐differentiation of contractile VSMCs.^[^ ^13^ ^]^ Specifically, KLF4 repressed the expression of Myocardin, providing a transcriptional mechanism for the fate‐switching of contractile VSMCs to a de‐differentiated state.^[^ ^14^, ^15^ ^]^ These changes resulted in the cells adopting a synthetic phenotype, characterized by increased proliferation and migration ability.^[^ ^16^ ^]^ Due to their remarkable phenotypic plasticity, VSMCs could adopt various cellular phenotypes, including those resembling foam cells, macrophages, mesenchymal stem cells, fibroblasts, and osteochondrogenic cells.^[^ ^17^, ^18^, ^19^ ^]^ This adaptability could influence the trajectory of plaque development and stabilization in both beneficial and detrimental ways. For instance, our previous study demonstrated that the deletion of peptidyl arginine deiminase 4 (PAD4) increased the proportion of fibroblast‐like cells derived from VSMCs.^[^ ^17^ ^]^ Additionally, VSMCs could trans‐differentiate into inflammatory macrophage‐like cells and calcified osteochondrogenic cells, contributing to the progression of vascular diseases.^[^ ^20^, ^21^ ^]^ Qing Zhu et al. found that the carotid artery balloon injury model induced VSMCs de‐differentiation and neointima formation in rats.^[^ ^22^ ^]^ Sun‐Hwa Song et al. also provided evidence of VSMCs de‐differentiation in injured human atherosclerotic plaque.^[^ ^23^ ^]^

However, it remains unclear whether de‐differentiated VSMCs can trans‐differentiate into fibroblast‐like cells that contribute to the repair of injured plaques or vessels. Additionally, the extent to which these fibroblast‐like cells facilitate the repair of disrupted plaques, as well as the signaling pathways regulating the trans‐differentiation of VSMCs into these cells, require further investigation.

To address this question, we utilized VSMCs lineage tracing mice, previously reported in our studies, and subjected them to a model of spontaneous plaque rupture.^[^ ^17^ ^]^ By integrating histology, single‐cell RNA sequencing (scRNA‐seq), and molecular analyses, we identified that TEAD1 could promote the trans‐differentiation of VSMCs into fibroblast‐like cells through the Wnt family member 4 (Wnt4)/β‐Catenin pathway, thereby promoting the healing process after plaque rupture.

## 2 Results

### 2.1 FSP1^+^ Cells were Expanded Along with Atherosclerosis Progression

**Figure 1:**
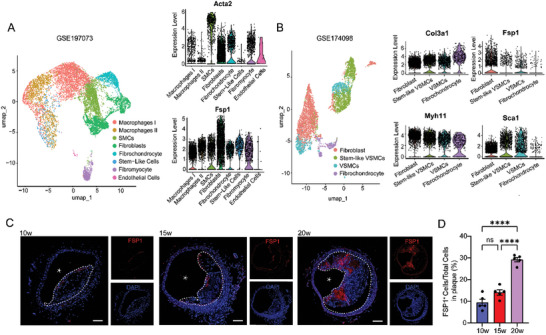
FSP1^+^ cells were expanded along with atherosclerosis progression. A,B) Uniform manifold approximation and projection (UMAP) visualization and violinplot of single cell RNA sequencing (scRNA‐seq) data from atherosclerotic plaques (GSE197073) and ligated carotid arteries (GSE174098). C) Immunofluorescence staining (IF) of FSP1 was performed on plaques from brachiocephalic artery of mice fed an HFD for 10, 15, and 20 weeks. The white dot line showed the plaque area, the white star represented lumen area. Scale bar = 100 µm. D) Quantification the percentage of FSP1^+^ cells/total cells in C (*n* = 5 per group). For all panels, error bars represented standard error of the mean (SE). *P*‐value was determined by one‐way ANOVA with Bonferroni post‐test (D). ns no significance, *P < 0.05, **P < 0.01, ***P < 0.001, and ****P < 0.0001.

To assess the involvement of fibroblast‐like cells in arterial remodeling, we first examined the fibroblast populations from the scRNA‐seq datasets of atherosclerotic plaques (GSE197073)^[^ ^17^ ^]^ and ligated carotid arteries (GSE174098).^[^ ^24^ ^]^ Our analysis revealed that cells expressing fibroblast markers Fibroblast Specific Protein 1 (FSP1), Collagen Type III Alpha 1 Chain (COL3A1) were abundant during the process of vascular injury (**Figure** **1**A,B). Furthermore, we stained FSP1 in plaques to distinguish their distribution and enrichment patterns during the development of atherosclerosis. Intriguingly, we observed an increased in FSP1^+^ (Fibroblast Specific Protein 1 positive) cells as plaque progression occurred, particularly in the fibrous cap region, which was identified using Masson staining (Figure 1C,D; Figure S1A, Supporting Information). Next, we analyzed carotid plaques from patients undergoing endarterectomy (GSE21545)^[^ ^25^ ^]^ and found that higher FSP1 expression within atherosclerotic plaque correlated with a better prognosis (Figure S1B, Supporting Information). These data indicated that the expansion of fibroblasts within advanced plaques might contribute to plaque stabilization.

### 2.2 VSMCs were the Primary Source of FSP1^+^ Cells in Atherosclerotic Plaques

**Figure 2:**
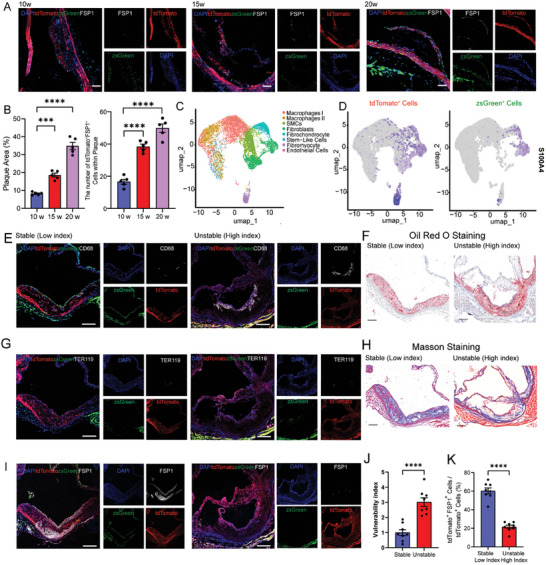
VSMCs were the primary source of FSP1^+^ cells in atherosclerotic plaques. A) IF staining of FSP1 on plaques from brachiocephalic artery of *Myh11^Cre^ B6G/R Ldlr^−/−^* mice fed an HFD for 10, 15, and 20 weeks. Scale bar = 100 µm. B) (Left) Quantification of the percentage of plaque area % (*n* = 5 per group). (Right) Quantification of the number of tdTomato^+^FSP1^+^ cells within the plaques harvested from three‐time points HFD‐fed *Myh11^Cre^ B6G/R Ldlr^−/−^* mice (*n* = 5 per group). C) UMAP visualization of our previous scRNA‐seq data. D) Featureplot of FSP1 (S100A4) on zsGreen^+^ fibroblasts and tdTomato^+^ fibroblasts from C. E) Representative images of CD68 on stable and unstable aortic root plaques. Scale bar = 100 µm. F) Representative images of Oil Red O staining on stable and unstable aortic root plaques. Scale bar = 100 µm. G) Representative images of TER119 on stable and unstable aortic root plaques. Scale bar = 100 µm. H) Representative images of Masson staining on stable and unstable aortic root plaques. Scale bar = 100 µm. I) Representative images of tdTomato^+^FSP1^+^ area on stable and unstable aortic root plaques. Scale bar = 100 µm. J) the quantification of vulnerability index between unstable (high index) and stable (low index) groups (*n* = 8 per group). K) the quantification of the percentage of tdTomato^+^ FSP1^+^ cells within plaque area between unstable (high index) and stable (low index) group (*n* = 8 per group). For all panels, error bars represented SE. *P*‐value was determined by unpaired two‐tailed Student's *t*‐test (J and K) or one‐way ANOVA with Bonferroni post‐test (B). ns no significance, *P < 0.05, **P < 0.01, ***P < 0.001, and ****P < 0.0001.

**Figure 3:**
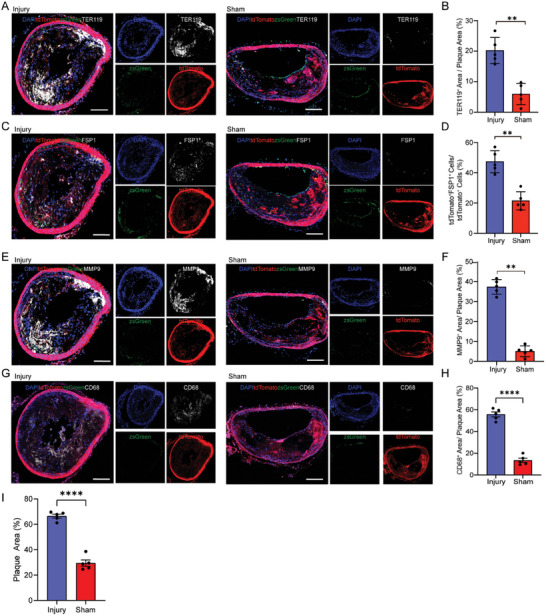
VSMCs‐derived FSP1^+^ fibroblast‐like cells contributed to healing after plaque rupture. A) Representative images of TER119 IF staining on brachiocephalic artery plaques harvested from *Myh11^Cre^ B6G/R Ldlr^−/−^* mice treated with either injury or sham surgery. Scale bar = 50 µm. B) Quantification of the percentage of TER119^+^ area/plaque area in A (*n* = 5 per group). C) Representative images of FSP1 IF staining on brachiocephalic artery plaques harvested from *Myh11^Cre^ B6G/R Ldlr^−/−^* mice treated with either injury or sham surgery. Scale bar = 50 µm. D) Quantification of the percentage of tdTomato^+^FSP1^+^ cells/tdTomato^+^ cells in C (*n* = 5 per group). E) Representative images of MMP9 IF staining on brachiocephalic artery plaques harvested from *Myh11^Cre^ B6G/R Ldlr^−/−^* mice treated with either injury or sham surgery. Scale bar = 50 µm. F) Quantification of the percentage of MMP9^+^ area/plaque area in E (*n* = 5 per group). G) Representative images of CD68 IF staining on brachiocephalic artery plaques harvested from *Myh11^Cre^ B6G/R Ldlr^−/−^* mice treated with either injury or sham surgery. Scale bar = 50 µm. H) Quantification of the percentage of CD68^+^ area/plaque area in G (*n* = 5 per group). I) Quantification of the percentage of plaque area % between injury and sham group. For all panels, error bars represented SE. *P*‐value was determined by unpaired two‐tailed Student's *t*‐test (B, D, F, H, and I). ns no significance, *P < 0.05, **P < 0.01, ***P < 0.001, and ****P < 0.0001.

Previous studies suggested that VSMCs, endothelial cells, and resident fibroblasts could give rise to FSP1^+^ cells within atherosclerotic plaques.^[^ ^26^, ^27^, ^28^ ^]^ However, the specific cell type that predominantly contributes to this population remains unknown. Given that VSMCs are the most abundant cell type within arteries and exhibit remarkable phenotypic plasticity during atherogenesis. We hypothesized that they might account for the majority of FSP1^+^ cells in atherosclerotic plaques.^[^ ^29^ ^]^ To test this hypothesis, we subjected VSMCs lineage tracing (*Myh11^Cre^ B6G/R Ldlr^−/−^*) mice to a high‐fat diet (HFD) and monitored the changes in FSP1^+^tdTomato^+^ cells within atherosclerotic plaques over 20 weeks. FSP1 staining demonstrated a progressive increase in the number of FSP1^+^tdTomato^+^ cells as plaque developed, with a primary localization within the fibrous cap region (**Figure** **2**A,B). Next, analysis of our previous scRNA‐seq dataset also confirmed that VSMCs were the predominant cell type capable of giving rise to fibroblast‐like cells in advanced plaques (Figure 2C,D). We next sought to explore whether FSP1^+^tdTomato^+^ cells were associated with plaque stability. The plaques of VSMCs lineage tracing mice were categorized into unstable (high vulnerability index) and stable (low vulnerability index) groups based on the median of vulnerability index, as previously described.^[^ ^30^, ^31^ ^]^ Briefly, the vulnerability index was calculated as a ratio of the sum of lipids (Oil red O, % plaque area), macrophages (CD68, % plaque area), and intra‐plaque hemorrhage (TER119, % plaque area) to the sum of smooth muscle cells (tdTomato^+^, % plaque area) and collagen (Masson Staining, % plaque area) (**Figure** **3**E–H). We then compared the percentage of VSMCs‐derived FSP1^+^ cells (FSP1^+^tdTomato^+^). We found that the percentage was decreased in plaques with a high vulnerability index compared to those with a low vulnerability index (Figure 3I–K). However, VSMCs could hardly trans‐differentiate into FSP1^+^ cells in the carotid arteries at 14‐ and 21‐days post‐ligation (Figure S2A,B, Supporting Information).

Next, we conducted a comparative analysis of VSMCs‐derived FSP1^+^ cells and fibromyocytes as characterized in Wirka's research.^[^ ^27^ ^]^ Utilizing scRNA‐seq from tdTomato^+^ VSMCs, we found that fibromyocytes exhibited higher expression of inflammatory markers, including Tumor Necrosis Factor Receptor Superfamily 11B（TNFRSF11B), Secreted Phosphorylation Protein 1 (SPP1), Tissue Inhibitor of Metallopeptidase 1 (TIMP1), and Lumican (LUM), compared to FSP1^+^ tdTomato^+^ cells. Despite this difference, both cell types could express fibroblast‐like markers such as Fibronectin 1 (FN1), and COL3A1 (Figure S2C–G, Supporting Information). In vitro investigations further confirmed that VSMCs‐derived FSP1^+^ cells induced by FGF21 showed low expression levels of LUM, TIMP1, and TNFRSF11b, while maintaining high levels of FN1, COL3A1, and FSP1 (Figure S2H, Supporting Information). Ingenuity Pathway Analysis (IPA) indicated that fibromyocytes might be linked to the secretion of inflammatory cytokines, whereas the genes activated in VSMCs‐derived FSP1^+^ cells were predominantly associated with signaling pathways related to plaque stability, particularly those involved in collagen secreting and ECM‐receptor interaction (Figure S2I, Supporting Information). So, we named this cell type as VSMCs‐derived FSP1^+^ fibroblast‐like cells. Furthermore, our investigation into their distribution during atheroprogression revealed that VSMCs‐derived FSP1^+^ fibroblast‐like cells (FSP1^+^tdTomato^+^ cells) were significantly more abundant than fibromyocytes (LUM^+^tdTomato^+^ cells) in atherosclerotic plaques of mice fed an High Fat Diets (HFD) for 16 weeks (Figure S3A,B, Supporting Information). Notably, VSMCs‐derived FSP1^+^ fibroblast‐like cells were predominantly located on the plaque surface, contrasting with the distribution of fibromyocytes (Figure S3A,C, Supporting Information). Additionally, we compared VSMCs‐derived FSP1^+^ fibroblast‐like cells with VSMC‐derived GLI1^+^ (GLI‐Kruppel family member GLI1 positive) adventitial progenitor cells recently (AdvSca1‐SMCs) described by Dubner.^[^ ^32^ ^]^ Our data showed a significantly lower number of AdvSca1‐SMCs, which were mainly localized within or at the base of atherosclerotic plaques, differing from the distribution pattern of VSMCs‐derived FSP1^+^ fibroblast‐like cells (Figure S3D–F, Supporting Information). This suggested that AdvSca1‐SMCs might not have a significant role in plaque stabilization and fibrous cap development. Taken together, our results indicated VSMCs served as the primary source of FSP1^+^ cells in atherosclerotic plaques, implicating these cells’ function in stabilizing atherosclerotic plaques.

### 2.3 VSMC‐Derived FSP1^+^ Fibroblast‐Like Cells Contributed to Healing after Plaque Rupture

Proteoglycans and type III collagen fibers are essential for the healing of plaques following rupture.^[^ ^33^ ^]^ Given that FSP1^+^tdTomato^+^ cells showed upregulation of the pathways related to collagen secretion (Figure S2I, Supporting Information), we hypothesized that these cells might be involved in the repair process after plaque rupture. To test this hypothesis, we simulated plaque rupture in ACS by externally clamping the plaques in the brachiocephalic artery of VSMC lineage tracing mice. Structural damage and increased TER119 staining in injured plaques, compared with sham plaques, provided evidence for the success of our plaque injury model (Figure 3A,B). Intriguingly, we observed a significant increase in FSP1^+^tdTomato^+^ cells within the injured plaques relative to the uninjured plaques (Figure 3C,D). Although the injury resulted in lager plaques with heightened inflammatory features, as indicated by elevated MMP9^+^ (Matrix Metalloproteinase‐9 positive) and CD68^+^ (Cluster of Differentiation 68 positive) areas (Figure 3E–I), the proportion of FSP1^+^tdTomato^+^ cells also increased significantly, indicating that plaque rupture coexisted with cellular repair processes. Collectively, these findings implied that VSMCs‐derived FSP1^+^ fibroblast‐like cells might contribute to healing after plaque rupture.

### 2.4 TEAD1 Promoted the Trans‐Differentiation of VSMCs into Fibroblast‐Like Cells and the Production of Extracellular Matrix

**Figure 4:**
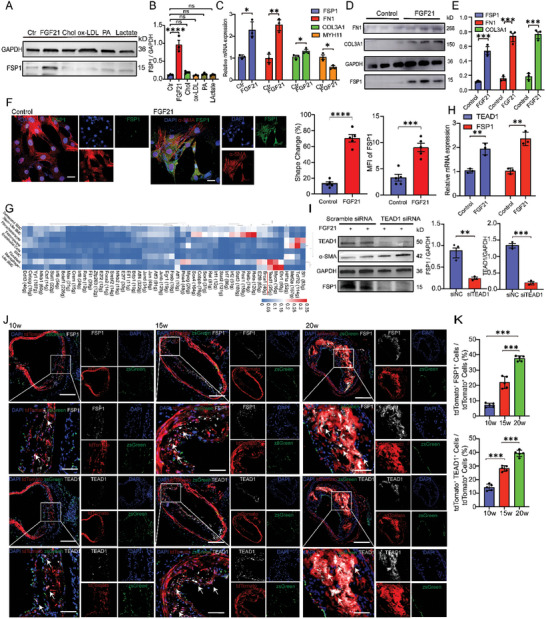
TEAD1 promoted the trans‐differentiation of VSMCs into fibroblast‐like cells and production of extracellular matrix. A) Representative WB images demonstrated that FGF21 exhibited the highest efficiency in inducing the trans‐differentiation of RASMCs into fibroblast‐like cells. B) Quantification of the WB results of A (*n* = 3 per group). C) The relative mRNA expression levels of FSP1, FN1, COL3A1, and MYH11 were compared between RASMCs and FGF21‐stimulated RASMCs (*n* = 3 per group). D) FGF21 promoted the expression of FSP1, FN1, and COL3A1 in RASMCs. E. Quantification of the WB results of D (*n* = 3 per group). F. The morphology changes in RASMCs following FGF21 stimulation by IF staining of FSP1 and α‐SMA (*n* = 5 per group). Scale bar = 20 µm. G) SCENIC analysis displayed the key transcriptional factors of different cell subtypes. H) The relative mRNA expression levels of TEAD1 and FSP1 were compared between RASMCs and FGF21‐stimulated RASMCs (*n* = 3 per group). I) After transfection with TEAD1 siRNA, the trans‐differentiation of RASMCs into fibroblast‐like cells was inhibited (*n* = 3 per group). J) IF staining of FSP1 and TEAD1 on the series plaques from brachiocephalic artery of *Myh11^Cre^ B6G/R Ldlr^−/−^* mice fed an HFD for 10, 15, and 20 weeks. The white arrows indicated tdTomato^+^FSP1^+^ cells or tdTomato^+^TEAD1^+^ cells. Scale bar = 100 and 20 µm. K) Quantification of the percentages of tdTomato^+^FSP1^+^ cells/ tdTomato^+^ cells and tdTomato^+^TEAD1^+^ cells/tdTomato^+^ cells in J (*n* = 5 per group). For all panels, error bars represented SE. *P*‐value was determined by unpaired two‐tailed Student's *t*‐test (C, E, F, H, and I) or one‐way ANOVA with Bonferroni post‐test (B and K). ns no significance, *P < 0.05, **P < 0.01, ***P < 0.001, and ****P < 0.0001.

After challenging rat aortic smooth muscle cells (RASMCs) with different stimulants,^[^ ^34^, ^35^, ^36^, ^37^ ^]^ we found that Fibroblast Growth Factor 21 (FGF21) had the strongest effect on inducing the expression of FSP1 (**Figure** **4**A,B). FGF21 treatment also upregulated the expression of FSP1, FN1, and COL3A1 in RASMCs (Figure 4C–E). Moreover, FGF21 induced a dramatic morphological shift in RASMCs, transitioning from the flattened, typical contractile phenotype of VSMCs to a spindle‐like, fibroblast‐like phenotype, along with increased FSP1 protein level (Figure 4F). Analysis of critical transcription factors involved in the trans‐differentiation of VSMCs into fibroblast‐like cells through Single‐cell Regulatory Network Inference and Clustering (SCENIC) analysis revealed TEAD1 expression in VSMCs‐derived fibroblast‐like cells was higher than other cell types (Figure 4G). Following FGF21 treatment, the messenger RNA (mRNA) expression of both TEAD1 and FSP1 was sharply upregulated (Figure 4H). Knockdown of TEAD1 via specific small interfering RNA (siRNA) in the presence of FGF21 stimulation resulted in a reduction of FSP1 protein expression by over 70% (Figure 4I). Furthermore, using adjacent series sections of plaques for TEAD1 and FSP1 staining, we noted that TEAD1 was localized in the same region of the fibrous cap where VSMCs‐derived FSP1^+^ cells were detected (Figure 4J,K). In human RNA‐sequencing (RNA‐seq) data from GSE21545, we also identified a positive correlation between TEAD1 and fibroblast markers (FSP1, FN1, and COL3A1). Conversely, TEAD1 expression was negatively associated with plaque instability‐related genes (MMP9 and Nod‐like Receptor Pyrin Domain Containing 3 (NLRP3)) (Figure S4A, Supporting Information). These findings implied a protective role for TEAD1 in promoting the trans‐differentiation of VSMCs into fibroblast‐like cells and stabilizing plaques. Several publications highlighted the critical role of TEAD1 in cellular function through interaction with its coactivators Yes‐Associated Protein (YAP) or Transcriptional Coactivator with PDZ‐binding motif (TAZ).^[^ ^38^, ^39^ ^]^ To explore their potential contribution to the TEAD1‐mediated mechanisms in VSMCs, we first assessed the expression changes of YAP or TAZ following FGF21 stimulation. We found that both YAP and TAZ were upregulated in RASMCs post‐stimulation. Moreover, both YAP and TAZ translocated into the nucleus and co‐localized with TEAD1 (Figure S4B,C, Supporting Information). Additionally, both the YAP inhibitor (Vertrporfin) and TAZ inhibitor (AR42) significantly inhibited the trans‐differentiation process in‐vitro, as evidenced by decreased the expression of fibroblast markers (COL3A1 and FSP1) in FGF21‐stimulated RASMCs (Figure S4D,E, Supporting Information). These results illustrated that TEAD1 was a putative critical regulator of VSMCs trans‐differentiation into fibroblast‐like cells, dependent upon the coactivators YAP and TAZ.

### 2.5 TEAD1 Promoted Plaque Stability and Healing Following Plaque Rupture via Mediating the Trans‐Differentiation of VMSCs into Fibroblast‐Like Cells

To explore the role of TEAD1 in the healing process after plaque rupture, we first used different concentrations of VT103 (0, 0.1, 1.0, and 10.0 µm) to inhibit its function in vitro. The results indicated that a concentration of 1.0 µm of VT‐103 effectively inhibited the trans‐differentiation of VSMCs into fibroblast‐like cells without adversely affecting the survival and apoptosis of other cell types (Figure S4F–L, Supporting Information). To further investigate its role in vivo, we induced spontaneous plaque rupture in atherosclerotic mice using a tandem stenosis (TS) model.^[^ ^40^ ^]^ Given that not all segments in the TS model could yield unstable plaques or experience plaque rupture, we employed MOVAT's Pentachrome staining, along with IF staining for Platelet Glycoprotein IIb (CD41), TER119, and MMP9 to identify the most appropriate segment for further analyses. As shown in Figure S5A–C (Supporting Information), all segments developed significant atherosclerotic lesions. After TS operations, atherosclerotic lesions in Segment 2 exhibited the largest necrotic area among these segments, as well as a higher density of type I collagen and a more loosely arranged type III collagen compared with the others. This implied increased plaque instability and a greater likelihood of rupture, followed by collagen generation during the repair process (Figure S5A,D–F, Supporting Information). Furthermore, the positive area of CD41, TER119, and MMP9 in Segment 2 exceeded those in other segments, suggesting intraplaque hemorrhage, thrombosis, and plaque instability, respectively (Figure S5G–I, Supporting Information). Therefore, we conducted further analyses based on lesions in Segment 2.

**Figure 5:**
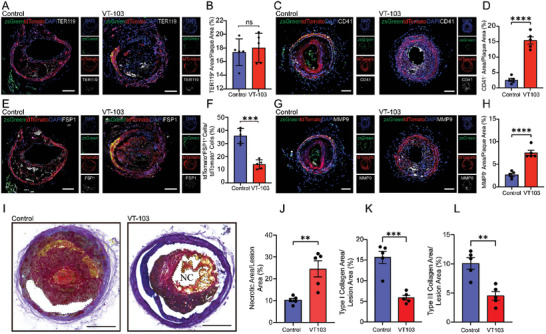
TEAD1 inhibitor VT‐103 attenuated plaque stability and hindered plaque healing after rupture. A,B) IF staining of TER119 on plaques from *Myh11^Cre^ B6G/R Ldlr^−/−^* mice that underwent TS surgery treated with either control or VT‐103 (*n* = 5 per group). Scale bar = 100 µm. C,D) IF staining of CD41 on plaques from *Myh11^Cre^ B6G/R Ldlr^−/−^* mice that underwent TS surgery treated with either control or VT‐103 (*n* = 5 per group). Scale bar = 100 µm. E,F) IF staining of FSP1 on plaques from *Myh11^Cre^ B6G/R Ldlr^−/−^* mice that underwent TS surgery treated with either control or VT‐103 (*n* = 5 per group). Scale bar = 100 µm. G,H) IF staining of MMP9 on plaques from *Myh11^Cre^ B6G/R Ldlr^−/−^* mice that underwent TS surgery treated with either control or VT‐103 (*n* = 5 per group). Scale bar = 100 µm. I) MOVAT pentachrome staining on plaques from *Myh11^Cre^ B6G/R Ldlr^−/−^* mice that underwent TS surgery treated with either control or VT‐103 (*n* = 5 per group). Scale bar = 100 µm. J–L) Quantification of the percentages of necrotic area/plaque area, type I collagen area/plaque area and type III collagen area/plaque area in I. For all panels, error bars represented SE. *P*‐value was determined by unpaired two‐tailed Student's *t*‐test (B, D, F, H, J, K, and L). ns no significance, *P < 0.05, **P < 0.01, ***P < 0.001, and ****P < 0.0001.

Next, the mice that underwent TS surgery were intraperitoneally injected with VT‐103 or control. Post‐surgery, TER119 staining was positive within the plaques, highlighting intraplaque hemorrhage, while it was hardly detected in plaques without surgical intervention (**Figure** **5**A,B; Figure S5J,K, Supporting Information). This finding confirmed that TS surgery successfully induced plaque injury. No significant differences in lipid profiles were observed between VT‐103 and control groups (Figure S5L, Supporting Information). However, the CD41^+^ area in ruptured plaques of mice treated with VT‐103 was significantly larger than that in control group, suggesting increased thrombosis and potentially repeated plaque rupture‐repair after TEAD1 inhibition (Figure 5C,D). Moreover, VT‐103 treatment decreased the percentage of VSMCs‐derived FSP1^+^ cells within lesions after TS surgery (Figure 5E,F), but did not impact the apoptosis of VSMCs (Figure S6A,B, Supporting Information). Additionally, plaque inflammation, assessed by MMP9 staining, was markedly elevated following VT‐103 treatment (Figure 5G,H). MOVAT pentachrome staining demonstrated that TEAD1 inhibition destabilized plaques and impeded their repair after rupture, as evidenced by an increased necrotic area and reduced collagen content within lesions after VT‐103 treatments (Figure 5I–L). As the function of TEAD1 was dependent upon the coactivators YAP and TAZ, we further explored their role in modulating healing after plaque rupture through intraperitoneal injection of YAP/TAZ enhancer GA‐017. We found that YAP/TAZ activation promoted the trans‐differentiation of VSMCs into FSP1^+^ fibroblast‐like cells (Figure S6C, Supporting Information), accompanied by reduced local inflammation and improved stability after plaque rupture, as demonstrated by decreased areas of MMP9, CD41, and TER119 following TS operations (Figure S6D–F, Supporting Information). These results illustrated that the TEAD1 could drive the trans‐differentiation of VSMCs into fibroblast‐like cells, thereby promoting plaque stability and plaque healing after rupture. We further observed the inhibition of trans‐differentiation of VSMCs into fibroblast‐like cells in mice without TS surgery (Figure S7A,B, Supporting Information) destabilized the plaques, resulting in thinner fibrous caps and decreased COL3A1 content (Figure S7C,D, Supporting Information). Additionally, VT‐103 increased the incidence of plaque erosion, as evidenced by heightened endothelial apoptosis and endothelial cell discontinuity (Figure S7E,F, Supporting Information). These results hinted to us that TEAD1 played a crucial role in facilitating plaque stability and healing following plaque rupture by mediating the trans‐differentiation of VMSCs into fibroblast‐like cells.

### 2.6 TEAD1 Promoted the Trans‐Differentiation of VSMCs into Fibroblast‐Like Cells via the Wnt4/β‐Catenin Signaling Pathway

**Figure 6:**
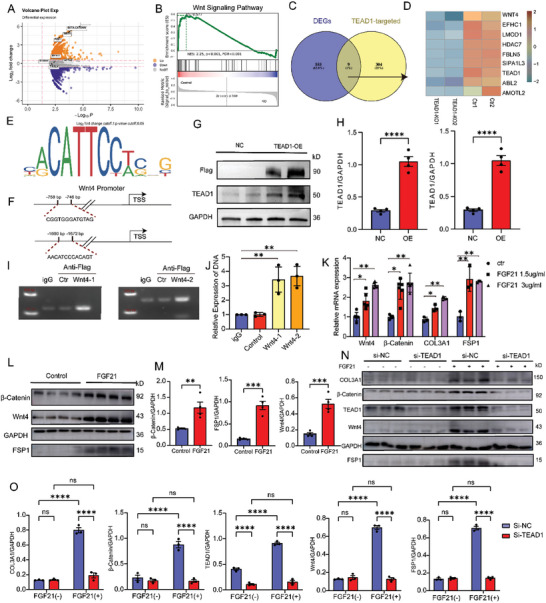
TEAD1 promoted the trans‐differentiation of VSMCs into fibroblast‐like cells via the Wnt4/β‐Catenin signaling pathway. A) Volcano plots of DEGs screened by comparing aortic media from TEAD1 KO and control group. B) GSEA was performed using the DEGs of A. C) The Venn diagram showed the DEGs from RNA‐seq and TEAD1‐targeted genes. D) The heatmap displayed the expression of the Wnt signaling pathway related genes. E,F) The predicted binding sites of TEAD1 on the Wnt4 promoter. G) WB results showed that RASMCs highly expressed TEAD1 and Flag protein after transfection with plasmid overexpression of TEAD1. H) Quantification of WB results of G (*n* = 4 per group). I) Gel electrophoresis analysis showed TEAD1 bound to the promoter region of Wnt4 after the TEAD1‐DNA complex was pulled using anti‐Flag antibodies. J) Quantification of results of I (*n* = 3 per group). K) The relative mRNA expression levels of Wnt4, β‐Catenin, COL3A1, and FSP1 were compared between RASMCs and RASMCs stimulated with FGF21 at concentrations of 1.5 and 3 µg ml^−1^ (*n* = 3 per group). L) WB results showed increased protein expression of Wnt4, β‐Catenin and FSP1 in RASMCs after FGF21 stimulation. M) Quantification of WB results of L (*n* = 4 per group). N) WB results indicated that the protein expression levels of TEAD1, Wnt4, β‐Catenin, FSP1, and COL3A1 were inhibited following transfection with TEAD1 siRNA in FGF21 stimulated RASMCs. O) Quantification of WB results of N (*n* = 3 per group). For all panels, error bars represented SE. *P*‐value was determined by unpaired two‐tailed Student's *t*‐test (H,M) or one‐way ANOVA with Bonferroni post‐test (J, K, and O). ns no significance, *P < 0.05, **P < 0.01, ***P < 0.001, and ****P < 0.0001.

To elucidate the mechanisms by which TEAD1 regulates the trans‐differentiation of VSMCs into fibroblast‐like cells, we analyzed the differentially expressed genes (DEGs) in the aortic media of the TEAD1 knockout group compared with control group through using a previously published RNA‐seq database (PRJNA492803). Gene Set Enrichment Analysis (GSEA) of the DEGs revealed significant enrichment in the Wnt signaling pathway (**Figure** **6**A,B). Based on these findings, we hypothesized that TEAD1 might regulate genes related to the Wnt4 signaling pathway. Further analysis of the RNA‐seq dataset identified specific DEGs regulated by TEAD1, indicating that TEAD1 primarily regulated genes of the Wnt4 signaling pathways (Figure 6C,D). Using the Just Another Sequence PASSword Archive (JASPAR) prediction algorithm (http://jaspar.genereg.net/), we identified a TEAD1 binding site in the promoter region of Wnt4 (Figure 6E,F). Overexpression studies of TEAD1 in RASMCs confirmed high protein expression of TEAD1 by WB (Figure 6G,H). Pulldown assays of the TEAD1‐DNA complex using CUT & Tag (Cleavage Under Targets and Tagmentation) qPCR, revealed that TEAD1 could bind to the promoter region of Wnt4 (Figure 6I,J). Therefore, TEAD1 might enhance Wnt4 expression by occupying its promoter region. Furthermore, stimulation with FGF21 in RASMCs significantly increased the expression of the Wnt4/β‐Catenin signaling pathway, which was accompanied by the trans‐differentiation of VSMCs into fibroblast‐like cells (Figure 6K–M). To evaluate the dependency of TEAD1 in mediating the activation of the Wnt4/β‐Catenin signaling pathway, siRNA knockdown studies demonstrated that TEAD1 deficiency reduced FGF21‐induced expression of Wnt4, β‐catenin, COL3A1, and FSP1 (Figure 6N,O). Taken together, these findings highlighted that TEAD1 promoted the trans‐differentiation of VSMCs into fibroblast‐like cells through the Wnt4/β‐Catenin signaling pathway.

### 2.7 Wnt4/β‐Catenin Signaling Pathway Mediated the Protective Role of TEAD1 in Healing Process after Plaque Rupture

**Figure 7:**
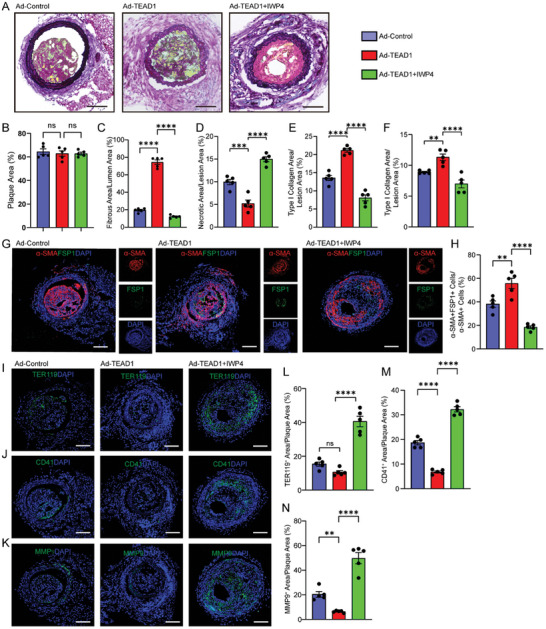
Wnt4/β‐Catenin inhibitor IWP4 could reverse the positive effect of SMC‐specific TEAD1‐AAV. A) Representative images of MOVAT pentachrome staining on plaques harvested from Ad‐control, Ad‐TEAD1 and Ad‐TEAD1+IWP4 groups. Scale Bar = 100 µm. B–F) Quantification the percentages of plaque area%, fibrous area%, necrotic area%, type I collagen area%, and type III collagen area% in A (*n* = 5 per group). G) IF staining of α‐SMA and FSP1 on plaques harvested from Ad‐control, Ad‐TEAD1 and Ad‐TEAD1+IWP4 groups. Scale bar = 100 µm. H) Quantification the percentage of α‐SMA^+^FSP1^+^ cells/α‐SMA^+^ cells in G (*n* = 5 per group). I–K) Representative IF staining of TER119, CD41 and MMP9 on plaques harvested from Ad‐control, Ad‐TEAD1 and Ad‐TEAD1+IWP4 groups. Scale bar = 100 µm. L–N) Quantification the percentages of TER119^+^ area/plaque area, CD41^+^ area/plaque area and MMP9^+^ area/plaque area in I–K, respectively (*n* = 5 per group). For all panels, error bars represented SE. *P*‐value was determined by one‐way ANOVA with Bonferroni post‐test. ns no significance, *P < 0.05, **P < 0.01, ***P < 0.001, and ****P < 0.0001.

We further investigated whether TEAD1's role in facilitating the trans‐differentiation of VSMCs into fibroblast‐like cells via the Wnt4/β‐catenin pathway in vitro similarly supported the reparative process after plaque rupture in vivo. After performing TS surgery, we injected the SMCs‐targeting TEAD1‐AAV three times to specifically overexpress TEAD1 in VSMCs of mouse arteries. No difference in plaque load was observed after TEAD1 overexpression (**Figure** **7**A,B). However, TEAD1 overexpression in VSMCs significantly enhanced the stability and healing after plaque rupture (Figure 7A,C–F), accompanied by an increase in VSMCs‐derived fibroblast‐like cells (α‐SMA^+^FSP1^+^ cells) (Figure 7G,H). As expected, the areas of TER119, CD41, and MMP9 were significantly reduced in mice injected with SMCs‐targeting TEAD1‐AAV (Figure 7I–N), indicating improved plaque repair and decreased plaque inflammation following rupture. Notably, the protective effects of TEAD1 overexpression in facilitating trans‐differentiation of VSMCs into fibroblast‐like cells and subsequent healing after plaque rupture were reversed by inhibition of Wnt4/β‐Catenin with IWP4 (Figure 7A–N). These results suggested that the Wnt4/β‐Catenin signaling pathway mediated the protective role of TEAD1 in the stabilization and repair of disrupted plaques. To further validate these findings, VSMC lineage tracing mice that underwent TS operations were locally injected with a Wnt4/β‐Catenin enhancer Norrin or PBS. Norrin nearly replicated the protective effects of SMCs‐targeting TEAD1‐AAV, as indicated by elevated tdTomato^+^FSP1^+^ cells and decreased areas of CD41, TER119, and MMP9 in disrupted plaques (Figure S8A–H, Supporting Information). Collectively, these results strongly suggested that TEAD1 promoted the trans‐differentiation of VSMCs into fibroblast‐like cells and the repair following plaque rupture through the Wnt4/β‐catenin signaling pathway.

## 3 Discussion

Plaque healing is crucial for maintaining the structure and stability of atherosclerotic plaques. Our study demonstrated that VSMCs could trans‐differentiate into fibroblast‐like cells, thereby contributing to plaque stabilization and healing following rupture. Inhibition of this transition destabilized plaques and impeded the healing process after rupture. Furthermore, we elucidated the role of TEAD1 in regulating the trans‐differentiation of VSMCs into fibroblast‐like cells via the Wnt4/β‐Catenin signaling pathway, which promoted plaque repair during the dynamic process of atherosclerosis. Our findings also provided insights into how the trans‐differentiation of VSMCs into fibroblast‐like cells contributed to maintaining a stable plaque structure and highlighted novel therapeutic targets for promoting atherosclerotic plaque healing after rupture.

Although plaque injury led to aggravated plaque inflammation, there was a rapid mobilization of VSMCs‐derived fibroblast‐like cells, suggesting that plaque rupture coexisted with the healing process. Our results might help explain the findings from a clinical study published by Yu et al.,^[^ ^41^ ^]^ which indicated that multilayered healing plaques reflected a larger scope and severity of the lesions. If plaque healing is insufficient to repair the rupture, the presence of local microthrombi and intraplaque hemorrhage (“first hit”) can increase plaque instability, resulting in a “second hit” as positive feedback.

Previous studies underscored the critical role of a range of immune cell types in plaque healing. For example, Back et al. demonstrated that the resolution of inflammation in ruptured plaques relied on M2 macrophages during the healing process.^[^ ^42^ ^]^ Similarly, the modulation of T‐cell activation and macrophages' phenotypic conversion also contributed to the plaque healing.^[^ ^43^ ^]^ Additionally, bone marrow‐derived endothelial cells could be recruited to participate in this process.^[^ ^44^ ^]^ While these studies mainly focused on exogenous and myeloid cells, the role of vascular resident cells in plaque repair remained poorly defined. Accumulating evidence showed that VSMCs in atherosclerotic plaques could alter their phenotype in response to different microenvironments, playing crucial roles in determining the pathological features of plaques.^[^ ^19^ ^]^ Our findings unveiled a novel role for VSMCs‐derived fibroblast‐like cells in promoting plaque stability and the healing process after rupture. We compared the VSMCs‐derived fibroblast‐like cells described here with the fibromyocytes reported by Wirka, et al.^[^ ^27^ ^]^ Compared with VSMCs‐derived fibroblast‐like cells, fibromyocytes exhibited greater proinflammatory properties and participated minimally in the healing process following plaque rupture (Figure S9A,B, Supporting Information). Regarding AdvSca1‐SMCs, as recently described by Dubner,^[^ ^32^ ^]^ we found that these cells were barely detectable after plaque rupture (Figure S9A,B, Supporting Information). This finding aligned with Zhou et al.’s conclusion that adventitial progenitor cells were mobilized for repair primarily in response to severe injuries (such as angiotomy), while minimal injuries (such as wire injury and atherosclerosis) yielded few detectable progenitor cells.^[^ ^45^ ^]^ Collectively, our finding supported that the VSMCs‐derived FSP1^+^ fibroblast‐like cells played a more significant role in plaque stabilization and repair process following plaque rupture.

As for mechanisms that regulated the trans‐differentiation of VSMCs into fibroblast‐like cells, we identified a key role of TEAD1 in this process. Several studies have investigated the function of TEAD1 in VSMCs in vascular injury models, such as femoral artery wire injury.^[^ ^46^, ^47^ ^]^ However, these studies primarily focused on its function in promoting VSMCs development and contractility. We also found that TEAD1 primarily regulated the transition of dedifferentiated VSMCs to fibroblast‐like cells rather than the de‐differentiation process (Figure S9C,D, Supporting Information). Our study revealed a novel mechanism by which TEAD1, coactivated by YAP/TAZ, upregulated the Wnt/β‐Catenin pathway, facilitating the trans‐differentiation into fibroblast‐like cells and the secretion of key extracellular matrix proteins, specifically COL3A1 and FN1.^[^ ^48^, ^49^ ^]^ These proteins were essential for initial repair process following plaque rupture.^[^ ^50^ ^]^ These results further substantiated the need for screening factors and mechanisms that promoted beneficial VSMCs phenotypic trans‐differentiation as potential therapeutic targets for improving plaque stability and post‐injury repair.

Previous studies have indicated that VSMCs can dedifferentiate into intermediate cells, and subsequently trans‐differentiated into multiple cell types, including macrophages, mesenchymal stem cells, fibroblasts, and osteochondrogenic cells, affecting the progression and stabilization of atherosclerotic plaques.^[^ ^51^ ^]^ Consequently, it has been proposed that therapeutic intervention for atherosclerosis should primarily focus on the inhibition of VSMCs dedifferentiation.^[^ ^52^, ^53^ ^]^ However, these studies ignored the potential benefits of promoting the trans‐differentiation of VSMCs into beneficial type cells, which could aid in plaque repair and stabilization. Our findings underscored the significance of this trans‐differentiation process, demonstrating that VSMCs converting into fibroblast‐like cells significantly contributed to stabilization by secreting extracellular matrix proteins such as COL3A1 and FN1. Our findings were consistent with previous studies showing VSMCs plasticity and its impact on plaque stability and repair. Furthermore, our study suggested that targeted modulation of these pathways could offer new therapeutic strategies for promoting stabilization and healing after plaque rupture.

We also recognized serval limitations in this study. Although the TEAD1 inhibitor, VT‐103, has been shown to effectively inhibit TEAD1‐mediated gene transcription, other cell types beyond VSMCs‐derived fibroblast‐like cells might also contribute to improving plaque stability and repair. To address this concern, we utilized a VSMCs‐specific AAV‐overexpression virus and observed improved repair following the specific overexpression of TEAD1 in VSMCs. While the dosage of the TEAD1 inhibitor could theoretically influence VSMCs trans‐differentiation and cell survival, we determined an optimal concentration of TEAD1 inhibitor in vitro that effectively inhibited the trans‐differentiation of VSMCs into fibroblast‐like cells without adversely affecting the survival or apoptosis of other cell types (Figure S4F–L, Supporting Information). Additionally, we noted clear differences and translational gaps in studying plaque rupture between mice and humans. These disparities were evident in hemodynamic alterations, shear stress, recruited immune and non‐immune cell types, inflammatory changes, and vessel integrity. The mouse TS plaque rupture model used in this study has been extensively characterized to elucidate how biological signaling pathways could impact plaque rupture.^[^ ^54^, ^55^ ^]^ Future studies that validate our findings under alternative experimental conditions or in larger animal models will be valuable.

In conclusion, the findings of this study established a foundation for innovative approaches aimed at promoting the repair and stability of injured atherosclerotic plaques through targeting VSMCs trans‐differentiation. Future research should focus on the development of targeted therapies, the validation of cell‐cell interaction, and the exploration of the broader implications of these mechanisms for clinical practice. This comprehensive approach will deepen our understanding of plaque stabilization and repair, potentially leading to effective new treatments for atherosclerosis.

## 4 Experimental Section

### Animals

All mice were housed in a controlled environment with a temperature maintained between 18 and 25 °C and a 12 h light/dark cycle. Male *Ldlr^−/−^*, *Myh11‐CreER^T2^ (Myh11^Cre^)* and *CAG‐LoxP‐ZsGreen‐Stop‐LoxP‐Tdtomato (B6G/R)* mice (purchased from Shanghai Model Organisms) were used in this study. *Ldlr^−/−^*, *Myh11^Cre^*, and *B6G/R* mice were genotyped by PCR based on the protocol provided by the company. In the *Myh11^Cre^ B6G/R Ldlr^−/−^* mouse models, Cre recombinase was activated in male mice with 75 mg kg^−1^ tamoxifen (T‐5648, Sigma, USA) intraperitoneal injections for 5 consecutive days at the age of 8 weeks. Male littermate controls were used for all studies. ZsGreen, located on the ROSA locus, was expressed in every single cell prior to tamoxifen induction. VSMCs and their progenies, which expressed Myh11, permanently expressed tdTomato following tamoxifen induction, resulting in a color change from green to red under fluorescent light. *Ldlr^−/−^* and *Myh11^Cre^ B6G/R Ldlr^−/−^* mice were fed a Clinton/Cybulsky High Fat Rodent Diet (HFD, 40% calories from fat; and 1.25% cholesterol, D12108C, Research Diets, USA) to induce atherosclerosis. Mice were sacrificed by cervical dislocation and perfused sequentially with 5 ml of PBS, followed by 10 ml of 4% paraformaldehyde, and finishing with another 5 ml of PBS via the left ventricle. Mouse aortas, brachiocephalic arteries, and hearts were carefully dissected, embedded in the Optimal Cutting Temperature compound, frozen, sectioned, and subjected to further analysis. For the tissue immunohistochemistry (IHC) analysis, the paraformaldehyde‐fixed samples were embedded in paraffin blocks and sectioned into 6 µm‐thick sections for subsequent experiments.

### Carotid Artery Ligation

Mice were anesthetized with intraperitoneal injection ketamine (80 mg kg^−1^) and xylazine (5 mg kg^−1^). The left carotid artery was completely ligated with a 6–0 silk suture proximal to the carotid bifurcation. The left carotid arteries were harvested for further analysis at 14‐ and 21‐day post‐ligation.

### Plaque Injury Model

The plaque injury model was developed to simulate the plaque rupture in patients with ACS, as initially described by Robert L et al.^[^ ^56^ ^]^ This method involves clipping the atherosclerotic plaque in the abdominal aorta of Apoe^−/−^ mice using round‐tipped forceps to induce plaque rupture. Minor modifications were implemented to standardize and enhance the reproducibility of this method in experiments. After anesthetizing *Myh11^Cre^ B6G/R Ldlr^−/−^* mice that had fed an HFD for 20 weeks, a partial sternotomy was performed to expose the brachiocephalic artery. Mechanical force was then applied to the brachiocephalic artery at the site of the plaque using hemostat forceps, maintaining pressure for 5 s intervals followed by 5 s intermissions until signs of plaque congestion were evident. Specifically, a resistive membrane pressure sensor was integrated at the force application point of the hemostat (Precision Kelly Hemostat, 5.5 Inches), allowing for precise maintenance of the pressure exerted on the blood vessel at ≈1000 g (as shown in Figure S9E, Supporting Information). A dedicated data acquisition device seamlessly collected the data from this sensor, enabling consistent monitoring of the pressure applied to the blood vessel, thereby enhancing the reproducibility and standardization of these experiments. After this procedure, the mice were fed with an HFD for an additional four weeks before sacrificed, at which point the artery plaque was harvested for further analysis.

### Tandem Stenosis (TS) Model of Plaque Rupture

The TS model was employed to induce spontaneous plaque rupture, as described previously.^[^ ^54^, ^55^ ^]^ Eight‐week‐old male mice were fed an HFD for 13 weeks prior to undergoing TS surgery. Following anesthetization, a small incision was made on the right common carotid artery, after which the tissue underneath was bluntly dissected. Two stenosis points were positioned 1 and 4 mm below the carotid bifurcation. A 150 µm needle was placed on the top of the right common carotid artery, and sutures were tightly tied at the two points to occlude blood flow. Subsequently, the needle was carefully removed to restore blood flow. After an additional seven weeks on HFD, the artery was harvested for further analysis.

### Cell Counting Kit‐8 (CCK‐8) Experiments

Cell viability was assessed by the CCK‐8 assay. Briefly, cells were seeded into a 96‐cell plate at a density of 2 × 10^3^ cells per well, with six replicates for each group. The cells were treated with different concentration of VT‐103. After 24 h, a 10 µl CCK8 reagent (HY‐K0301, MCE, USA) was added according to the manufacturer's instructions, followed by a 2 h incubation. The absorbance was measured at 450 nm using a microplate reader.

### Atherosclerotic Lesion Analyses

The plaques from aortic root, right common carotid arteries, and brachiocephalic artery were processed for histology. The fibrous cap was identified using Masson staining. MOVAT pentachrome staining was performed using a commercial staining kit (S191078, Pinuofei Biological, China) according to the manufacturer's instructions. Briefly, sections were incubated in Weigert's iron hematoxylin for 10 min to stain nuclei. After differentiation in acid alcohol, collagen appeared pink. Subsequently, sections were stained in Biebrich Scarlet‐Acid Fuchsin solution for 15 min to highlight elastic fibers and muscle. Differentiation in phosphotungstic/phosphomolybdic acid solution for 10 min emphasized the distinction between collagen and ground substance. Aniline Blue staining for 10 min highlighted glycosaminoglycans. Stained sections were examined under a light microscope equipped with polarizing filters to optimize visualization of collagen and elastic fibers. Digital micrographs were captured at representative magnifications for subsequent analysis. Plaque area, necrotic core, type I and type III collagen content, and other relevant parameters were quantified using image J software (Version 2.10, NIH, USA). As for the plaque area% calculation, it was accurately assessed through dividing the plaque area by the internal elastic lamina (IEL) area.

### Cell Culture and Stimulation

Rat aortic smooth muscle cells (A7r5, RASMCs) were purchased from Shanghai Zhongqiaoxinzhou Biotechnology. The cells were cultured in DMEM (SH30243.01, HyClone, USA) supplemented with 10% fetal bovine serum (FBS) (10 099 141, Gibco, USA) and 1% penicillin‐streptomycin at 37 °C in an atmosphere of 95% O_2_ and 5% CO_2_. To induce the trans‐differentiation of RASMCs into fibroblast‐like cells, the RASMCs were starved in DMEM with 0.2% FBS overnight and subsequently stimulated with FGF21 (1.5 ng ml^−1^, HY‐P7173, MCE, USA) for 48 h. In some experiments, the RASMCs were stimulated with water‐soluble cholesterol at concentration of 10 µg ml^−1^ for 72 h (C4951‐30MG, Sigma, USA), ox‐LDL at concentration of 100 µg ml^−1^ for 48 h (P00794, Solarbio, USA), palmitic acid at concentration of 300 µmol L^−1^ for 12 h (HY‐N0830, MCE, USA), lactate at concentration of 10 mm for 12 h (HY‐B227, MCE, USA). To further investigate the roles of YAP and TAZ in the trans‐differentiation of VSMCs, the YAP inhibitor Verteporfin (10 µg ml^−1^, SML0534, Sigma, USA), and the TAZ inhibitor AR42 (600 nm, T6392, Target Molecule Corporation, USA) were used for treatment. The specific procedure was as follows: under FGF21 stimulation, RASMCs were treated with either Verteporfin or AR42 for 12 h. The experimental groups included: control group (Ctr), FGF21‐only stimulation group (FGF21), FGF21 stimulation with Verteporfin treatment group, and FGF21 stimulation with AR42 treatment group. The effects of YAP and TAZ inhibition on the trans‐differentiation of RASMCs into fibroblast‐like cells induced by FGF21 were evaluated by examining the expression changes of trans‐differentiation related markers in the different treatment groups. Human umbilical vein endothelial cells (HUVECs) were cultured in Ham's Kaighn's Modification F12K medium (Invitrogen, USA) supplemented with 10% FBS, 1% penicillin‐streptomycin, heparin (15 IU ml^−1^, LEO Pharma, Denmark) and vascular endothelial growth factor (30 ng ml^−1^, Sigma, USA). NIH‐3T3 fibroblast cells were grown in DMEM supplemented with 10% FBS and 1% penicillin‐streptomycin. After stimulations, the cells were harvested for downstream experiments.

### In Vivo Treatments with TEAD1 Inhibitor (VT‐103), YAP/TAZ Enhancer (GA‐017), and Wnt4/β‐Catenin Enhancer (Norrin)

VT‐103 was administered intraperitoneally into mice at a dosage of 0.3 mg kg^−1^ three times per week for seven weeks following TS surgery, until the animals were sacrificed. In the in vivo injection experiments involving the YAP/TAZ enhancer (GA‐017), GA‐017 was injected intraperitoneally at a dosage of 0.1 mg kg^−1^ three times per week after TS surgery for seven weeks until sacrifice. For the local injection of Wnt4/β‐Catenin enhancer Norrin, atherosclerotic mice underwent TS operation, followed by a local injection of either the Wnt4/β‐Catenin enhancer Norrin (250 ng, 3497‐NR, Biotechne, USA) or PBS as a negative control prior to the closure of the incision. After seven weeks, both groups of mice were sacrificed for further experiments and analysis.

### Intravenous Injection of Adeno‐Associated Virus (AAV) and IWP4 Treatments

AAV targeting mouse TEAD1 (Ad‐TEAD1) and an empty vector (Ad‐Control) were obtained from GENECHEM Incorporation (Shanghai, China). At the time of TS surgery, the Sm22ap‐MCS‐SV40 AAV virus was intravenously injected at a dosage of 0.5 × 10^12^ viral genomes (v.g) per mouse. Two days after AAV virus injection, a group of mice received Ad‐TEAD1 were administered with the Wnt4/β‐Catenin inhibitor IWP4 (5214, Biotechne, USA) intraperitoneally at a dosage of 4 mg kg^−1^ per day for 10 days. After seven weeks on an HFD, the mice were sacrificed for subsequent analysis.

### Small Interfering RNA (siRNA) Transfection

siRNA targeting TEAD1 (siTEAD1) and scramble siRNA (siNC) were purchased from GenePharma (China). The RASMCs were transfected with 50 nm siRNA using Lipofectamine iMAX reagent (13 778 030, Thermo Fisher, USA) for 8 h before downstream experiments were performed. The sequences of siRNAs are shown in Table S1 (Supporting Information).

### Plasmid Construction and Transfection

TEAD1‐Flag was constructed and purchased from Shanghai Genechem Co, Ltd. The plasmids were transfected into RASMCs at a concentration of 20 µg ml^−1^ using Lipofectamine iMAX reagent (13 778 030, Thermo Fisher, USA) for 6 h.

### Immunofluorescence (IF) Staining and Laser Confocal Fluorescence Microscopy Analysis

Cells were seeded at 1 × 10^6^ cells per well on a glass‐bottomed culture dish and fixed in 4% paraformaldehyde for 15 min, followed by permeabilization in 0.2% Triton X‐100 (X100RS, Sigma, USA) in PBS for 5 min. Cross sections were also fixed in 4% paraformaldehyde for 15 min and permeabilized in 0.2% Triton X‐100 in PBS for 5 min. Permeabilized cells or tissue sections were washed three times with PBS and then blocked with 5% goat serum. The sections or cells were incubated with primary antibodies diluted in a blocking buffer, including anti‐FSP1 (1:100, ab124805, Abcam, USA), anti‐CD31 (1:100, ab222783, Abcam, USA), anti‐TER119 (1:100, 116 202, Biolegend, USA), anti‐MMP9 (1:100, ab38898, Abcam, USA), anti‐TEAD1 (1:100, ab133533, Abcam, USA), anti‐α‐SMA (1:200, ab7817, Abcam, USA), anti‐LUM (1:250, ab168348, Abcam, USA), anti‐GLI1 (1:100. sc‐515781, Santa Cruz, USA), anti‐CD41 (1:100, sc‐373992, Santa Cruz, USA), anti‐CD68 (1:100, NB100‐683, Novus, USA). Normal isotype IgG (1:100, sc2027, Santa Cruz, USA) was used as a negative control. After three washes with PBS, cells or tissue sections were incubated with secondary antibodies Alexa Fluor 647‐conjugated goat anti‐rabbit (1:200, A‐21244, Invitrogen, USA), Alexa Fluor 488‐conjugated goat anti‐rabbit (1:200, A‐11008, Invitrogen, USA), Alexa Fluor 594‐conjugated donkey anti‐rabbit (1:200, R37119, Invitrogen, USA) or Alexa Fluor 594‐conjugated goat anti‐mouse (1:200, A‐11005, Invitrogen, USA) for 1 h at 37 °C in the dark. Nuclei were labeled with DAPI (Vector Laboratories), and cells were visualized using an LSM710 laser confocal microscope (Carl Zeiss, Germany). A close examination of each plane of the z‐stack was conducted using Zen 2009 Light Edition Software (Zeiss) to confirm that the presence of immunofluorescence staining coincided with a single DAPI nucleus. Image J was used to quantify the lesion area or the positive region.

### Immunohistochemistry (IHC) Analysis

For IHC, antigen retrieval was performed after paraffin sections were dewaxed and rehydrated. Then tissue sections were then blocked with 5% goat serum and incubated with primary antibodies targeting α‐SMA (1:200, ab7817, Abcam, USA), CD68 (1:100, NB100‐683, Novus, USA), and COL3A1 (1:100, sc‐8784, Santa Cruz, USA) at 37 °C for 1 h. After three washes with PBS, the sections were incubated with secondary antibodies at 37 °C for 1 h. The DAB Horseradish Peroxidase Color Development Kit (DA1015, Solarbio, China) was used to visualize the positive signals in the sections. Cell nuclei were counterstained with Mayer's hematoxylin solution. Image J was used to calculate the ratio of the positive area of α‐SMA, CD68, or COL3A1 relative to the total plaque area.

### TUNEL Assay

TUNEL assay was used to evaluate apoptotic cells according to the manufacturer's instruction (40307ES20, YEASEN, China). Image J was used to quantify TUNEL‐positive cells in each sample under 20 × magnification.

### Protein Extraction and Western Blotting (WB)

Whole cell lysate was prepared by 1 × cell lysis buffer (9803, Cell Signaling Technologies, USA) with protease inhibitors (0 469 315 9001, Roche Molecular Biochemicals, USA) and phosphatase inhibitor (78 428, ThermoFisher Scientific, USA). Protein concentrations were determined by using a BCA protein assay kit. Proteins were separated by SDS–PAGE, transferred to polyvinylidene fluoride (PVDF) membranes, and incubated with antibodies diluted with a blocking buffer overnight at 4 °C, including anti‐FSP1 (1:1000, ab124805, Abcam, USA), anti‐FN1 (1:500, sc‐8422, Santa Cruz, USA), anti‐COL3A1 (1:500, sc‐514601, Santa Cruz, USA), anti‐TEAD1 (1:1000, ab133533, Abcam, USA), anti‐α‐SMA (1:1000, ab7817, Abcam, USA), anti‐β‐Catenin (1:1000, ab32572, Abcam, USA), anti‐Wnt4 (1:500, sc‐376279, Santa Cruz, USA), and anti‐GAPDH (1:5000, 60004‐1‐Ig, Proteintech, USA). Then the PVDF membranes were incubated with HRP‐conjugated secondary antibodies for 1 h and bands were visualized using chemiluminescence (TANON, China) and viewed under an Amersham Imager 600 system (GE Health‐care, USA).

### Real‐Time Quantitative RT‐PCR

Total RNA was extracted using Trizol regent (Thermo Fisher, USA) according to the manufacturer's instructions. Purified RNA (1000 ng) was reverse‐transcribed using a Primer Script RT Reagent Kit (#rr820a, Takara, Japan). The quantitative RT‐PCR was then performed on 1 µg of cDNA product using FastStart Universal SYBR Green Master (QR0100, Roche, USA) on a Roche Light cycler. The RT‐PCR primers are listed in the Table S2 (Supporting Information).

### ScRNA‐Seq Data Processing

The scRNA‐seq data for tdTomato^+^ cells and zsGreen^+^ cells from the aortas of HFD‐fed *Myh11^Cre^ B6G/R* mice (GSE197073) were generated in the previous study.^[^ ^17^ ^]^ In addition, a publicly available scRNA‐seq dataset of the carotid artery ligation model (GSE174098) was downloaded from the GEO database. The datasets were analyzed using Seurat v3.1.1 in R. The *Myh11^Cre^Padi4^flox/flox^* part of scRNA‐seq were excluded from this data for further analysis. Quality control was performed to exclude cells with poor gene expression profiles, specifically those expressing fewer than 200 genes, or those with more than 10% mitochondrial genes among the total expressed genes. After normalization, Principal Component Analysis was performed and calculated the number of significant principal components using the Elbow Plot function. The Uniform manifold approximation and projection (UMAP) was performed for unsupervised clustering. The resolution was set to 0.6 because it allows for a better annotation of cell types. In addition, marker genes were defined based on the Find Marker function and literature.

### SCENIC Analysis

The R package SCENIC (https://github.com/aertslab/SCENIC, version 1.1.1‐10, RcisTarget 1.6.0, and AUCell 1.8.0) was used to analyze the enrichment of transcriptome factors in different cell subtypes.^[^ ^57^ ^]^ The input matrices in SCENIC were the raw UMI counts obtained from Seurat. Following the standard SCENIC procedure, the GENIE3 method was used to identify potential transcription factor targets. In addition, the activity of each regulon in each cell was evaluated using AUCell, calculated as the area under the recovery curve, which integrated the expression ranks across all genes within a regulon.

### Differential Expression and Pathway Enrichment Analysis

DEGs of cell subtypes were identified using Find Markers in the Seurat package with the Wilcoxon test. |Log_2_(FC)|>1 and adjusted *P* value < 0.05 were set as the cut‐off criteria. Pathway enrichment analysis was performed using QIAGEN‐Ingenuity Pathway Analysis (IPA) software (www.ingenuity.com). The Fisher's Exact Test was used to calculate a statistical significance of the overlap of differentially expressed transcripts with canonical pathways, and pathways were selected based on statistical significance (*P* value<0.05) and enrichment (Z) score. All analyses were performed in R v4.3.3 statistical environment and the plots were generated with ggplot2 v3.5.1 library.

### The Association between Biomarkers and Prognosis in Bulk RNA‐Seq Dataset

The bulk RNA‐seq and prognostic data were downloaded from GSE21545 dataset. The Kaplan‐Meier curves were plotted based on the median expression levels of TNF‐α, FSP1, SPP1, and ACAT2 in R v4.3.3 statistical environment.

### Bulk RNA‐Seq and Bioinformatics Analysis

Bulk RNA‐Seq dataset (PRJNA492803) for the aortic media from the TEAD1 CKO group and control group mice was downloaded from NIH BioProject. The CKO group utilized an inducible smooth muscle–specific TEAD1 KO (knockout) mouse model. DEG analysis was performed using the limma R package. Genes with log_2_FC ≥1 and adjusted *P* value <0.05 were considered as significant DEGs. Subsequently, Gene Set Enrichment Analysis (GSEA) was conducted using the “GSEA” package to reveal signaling pathways associated with these significant DEGs.

### CUT & Tag PCR

With minor modifications, CUT & Tag was performed using the Hyperactive Universal CUT & Tag Assay Kit for Illumina (TD903, Vazyme, China). A total of 1 × 10^5^ cells were gently washed twice with PBS. Then 10 µl of concanavalin A‐coated magnetic beads were added to each sample and incubated at room temperature for 10 min. After discarding the unbound cells, the bound cells were mixed with an anti‐Flag antibody (1:50, 14 793, CST, USA) or rabbit normal IgG antibody (1:200, 2729, CST, USA) and incubated at 4 °C overnight with rotation. Next, cells were incubated in a secondary antibody, diluted in DIG wash buffer, at room temperature for 1 h, and washed three times with DIG wash buffer. The beads were then incubated with a pA/G‐Tnp adapter complex (0.04 µm) at room temperature for 1 h with rotation and washed three times with DIG‐300 buffer. DNA was purified using phenol‐chloroform isoamyl alcohol extraction and ethanol precipitation. To amplify the library, 15 µl of purified DNA was mixed with 5 µl of a universal i5 and a uniquely barcoded i7 primer. A volume of 25 µl 2 × CUT & Tag amplification mix was added and mixed. The samples were placed in a PCR thermal cycler with a heated lid using the following program: 72 °C for 3 min; 95 °C for 3 min; 18 cycles of 98 °C for 10 s and 60 °C for 5 s; final extension at 72 °C for 1 min and hold at 4 °C. PCR products were purified using VAHTS DNA Clean Beads (N411‐01, Vazyme, China).

### Statistics

Statistical analyses were performed using GraphPad Prism (Version 9.0). Results were displayed as mean ± standard error of the mean (SE). Student's *t*‐test or Mann‐Whitney *U*‐test was used to analyze the difference between standard and non‐normal variables. Differences across more than three groups were compared using one‐way ANOVA followed by a post hoc analysis with the Bonferroni test. Two‐sided and *p *< 0.05 was regarded as statistical significance.

## Conflict of Interest

The authors declare no conflict of interest.

## Author Contributions

M.Z., Z.L., and Y.S. contributed equally to this work. W.P., M.W.F., Q.Y., J.Z., and W.J. conceived and designed the study and experiments. M.Z., Z.L., and J.S. performed the experiments and data analyses. Y.S. and Q.Y. supervised the study. M.Z., Z.L., Y.Z., and S.G. interpreted the data and wrote the manuscript. All authors revised, critically discussed, and approved the final version of the manuscript.

## Supporting information



Supporting Information

Supplemental Table 1

Supplemental Table 2

## Data Availability

The data that support the findings of this study are available from the corresponding author upon reasonable request.
