# Supplementary material for: TEAD1‐Mediated Trans‐Differentiation of Vascular Smooth Muscle Cells into Fibroblast‐Like Cells Contributes to the Stabilization and Repair of Disrupted Atherosclerotic Plaques
Source: Adv Sci (Weinh). 2024 Dec 12;12(5):2407408. doi: 10.1002/advs.202407408 (PMC11791998; doi:10.1002/advs.202407408)
Supplement: Supplementary file 1 — Supporting Information [file ADVS-12-2407408-s003.docx]

**
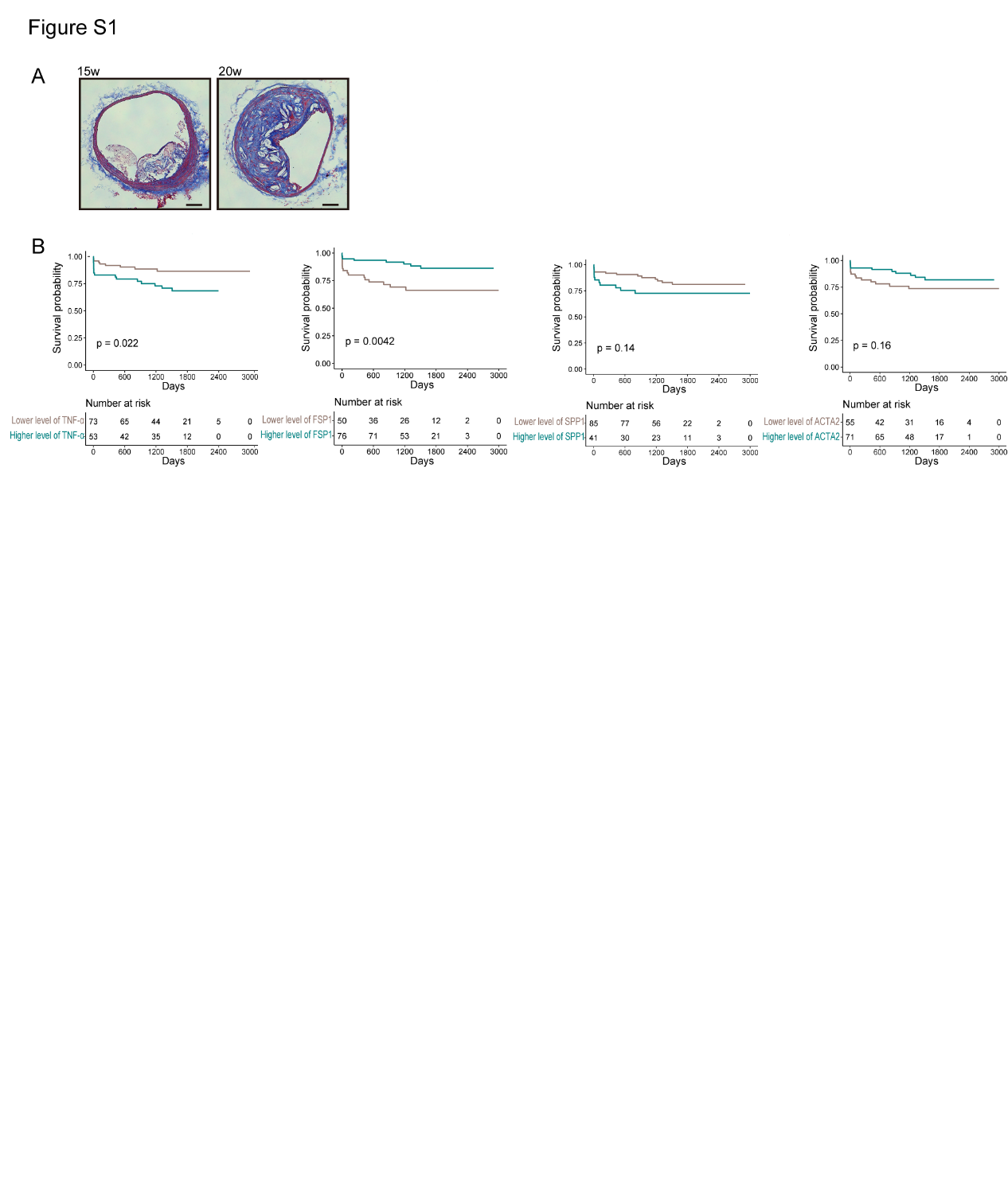
**

**Figure S1.** **Human RNA-seq data revealed the association between cell biomarkers and clinical prognosis**. **A**. The representative images of Masson staining in **Figure 1C**. **B**. Kaplan-Meier curves for TNF-α, FSP1, SPP1 and ACTA2 in patients underwent endarterectomy from GSE21545. The *P*-value was determined by Log-rank test.


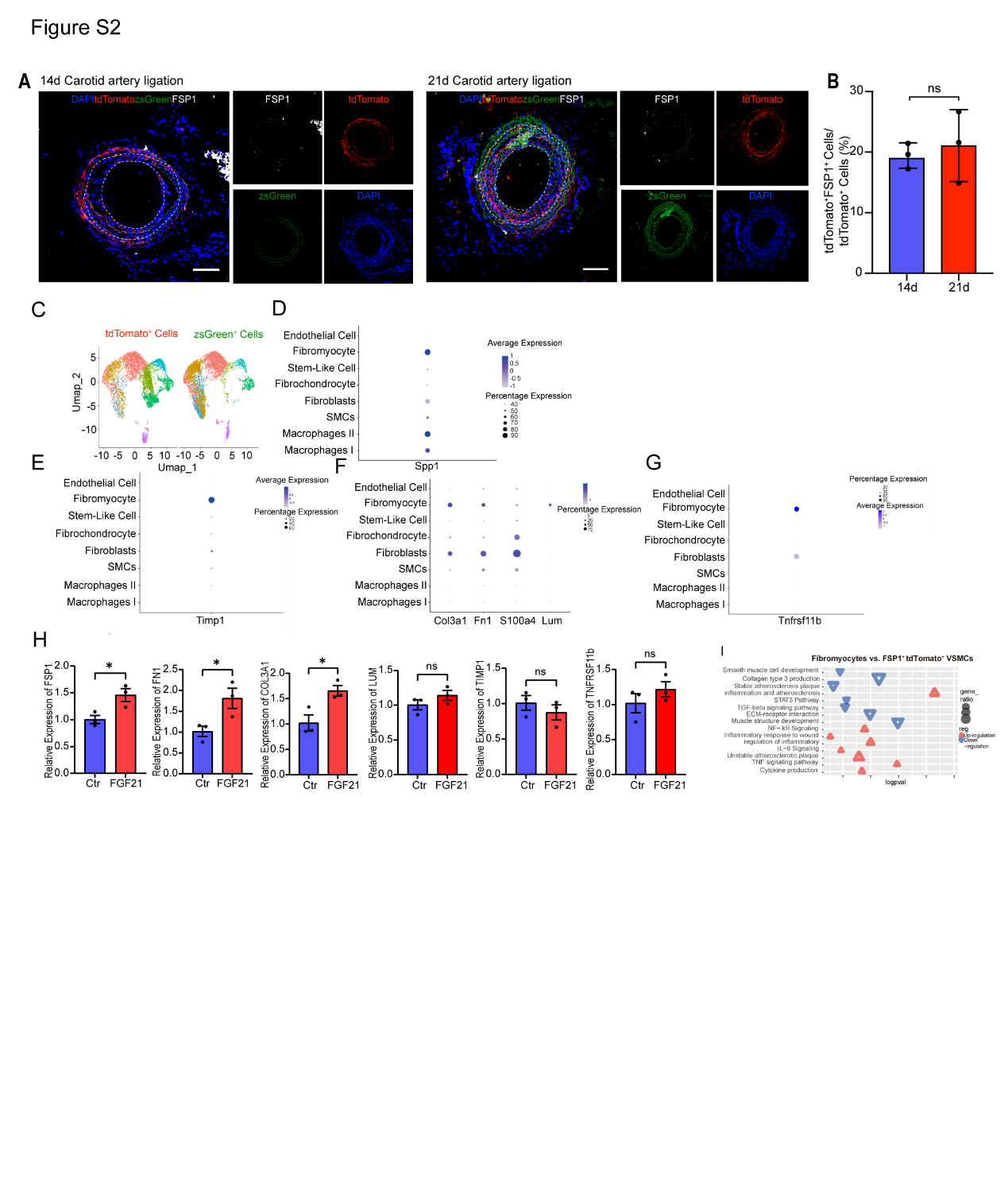


**Figure S2.** **Representative FSP1 IF staining on** **carotid artery after ligation. Differential expression and pathway enrichment analysis of different sources of fibroblast. A**. IF staining of FSP1 on carotid artery after 14 days and 21 days of ligation in *B6G/RMyh11^Cre^* mice. Scale bar=100μm **B**. Quantification the percentage of tdTomato^+^FSP1^+^ cells/tdTomato^+^ cells in **A** (n=5 per group). **C**. UMAP visualization of the tdTomato^+^ VSMCs and zsGreen^+^ non-VSMCs cells in our previously scRNA-seq (GSE197073). **D, E, F and G.** Dotplots showed the expression of SPP1, TIMP1, COL3A1, FN1, S100A4, LUM and TNFRSF11b in different phenotypic transdifferentiated cells derived from VSMCs. **H**. The relative mRNA expression levels of several markers in VSMCs-derived fibroblast-like cells in vitro (n=3 per group). **I**. IPA analysis of DEGs screened from fibromyocytes vs. FSP1^+^tdTomato^+^ cells. For all panels, error bars represented SE. *P*-value was determined by unpaired two-tailed Student’s *t*-test (**H**). ns no significance, * *P* <0.05, ** *P* <0.01, *** *P* <0.001, **** *P* <0.0001.

**
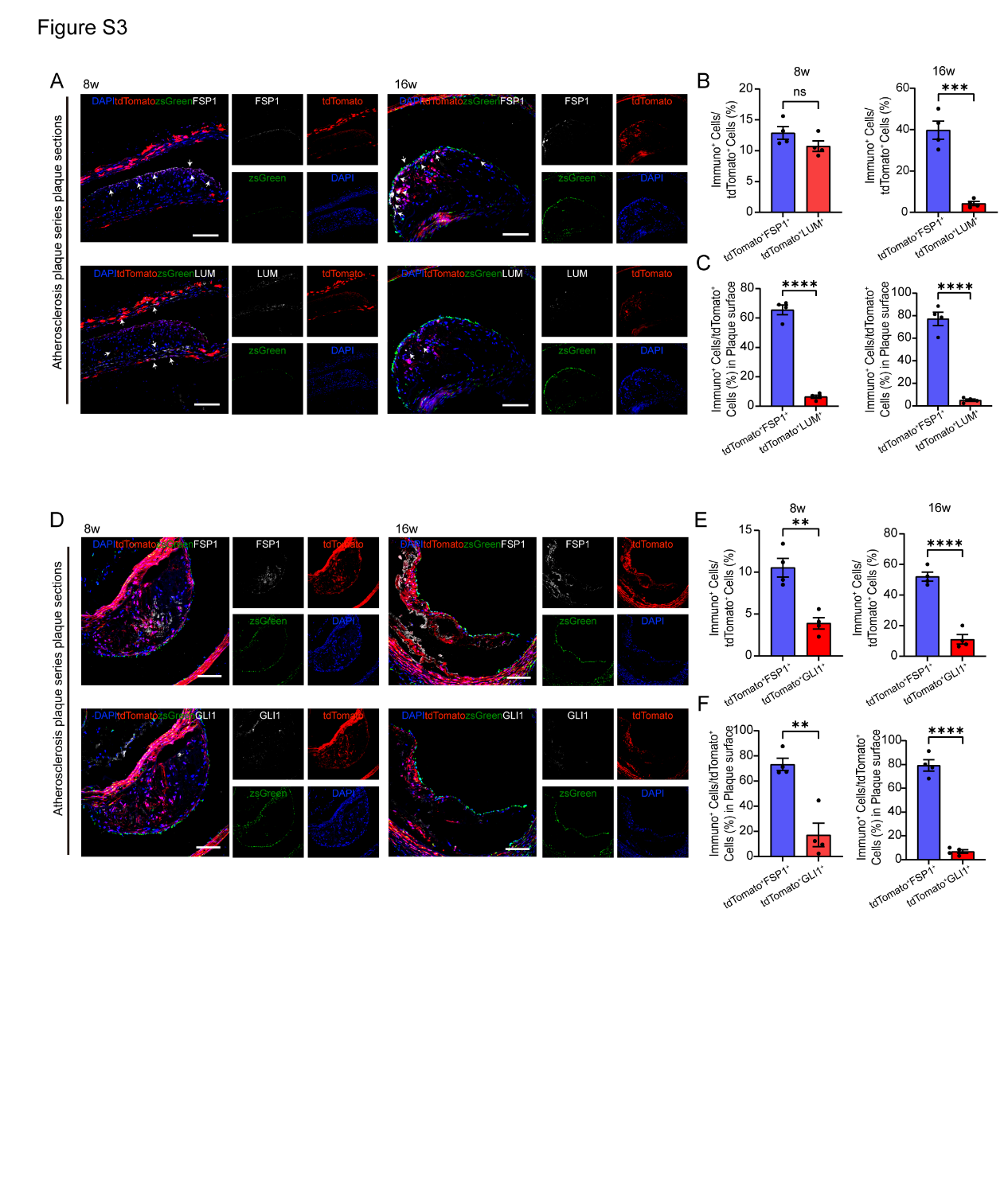
**

**Figure S3.** **Representative IF staining of FSP1, LUM and GLI1 on plaques harvested from mice fed an HFD for 8 and 16 weeks. A**. Representative IF staining of FSP1 and LUM on plaques harvested from *Myh11^Cre^ B6G/R Ldlr^-/-^* mice fed an HFD for 8 and 16 weeks. The white arrows indicated tdTomato^+^FSP1^+^ cells or tdTomato^+^LUM^+^ cells. Scale bar =50μm. **B**. Quantification of the percentages of tdTomato^+^FSP1^+^ cells/tdTomato^+^ cells and tdTomato^+^LUM^+^ cells/tdTomato^+^ cells within plaques during the atherosclerosis progression (n=5 per group). **C**. Quantification of the percentages of tdTomato^+^FSP1^+^ cells/tdTomato^+^ cells and tdTomato^+^LUM^+^ cells/tdTomato^+^ cells within plaque surface during the atherosclerosis progression (n=5 per group). **D**. Representative IF staining of FSP1 and GLI1 on plaques harvested from *Myh11^Cre^ B6G/R Ldlr^-/-^* mice fed an HFD for 8 and 16 weeks. Scale bar =50μm. **E**. Quantification of the percentages of tdTomato^+^FSP1^+^ cells/tdTomato^+^ cells and tdTomato^+^GLI1^+^ cells/tdTomato^+^ cells within plaques during the atherosclerosis progression (n=5 per group). **F**. Quantification of the percentages of tdTomato^+^FSP1^+^ cells/tdTomato^+^ cells and tdTomato^+^GLI1^+^ cells/tdTomato^+^ cells within plaque surface during the atherosclerosis progression (n=5 per group). For all panels, error bars represented SE. *P*-value was determined by unpaired two-tailed Student’s *t*-test (**B, C, E and F**). ns no significance, * *P* <0.05, ** *P* <0.01, *** *P* <0.001, **** *P* <0.0001.


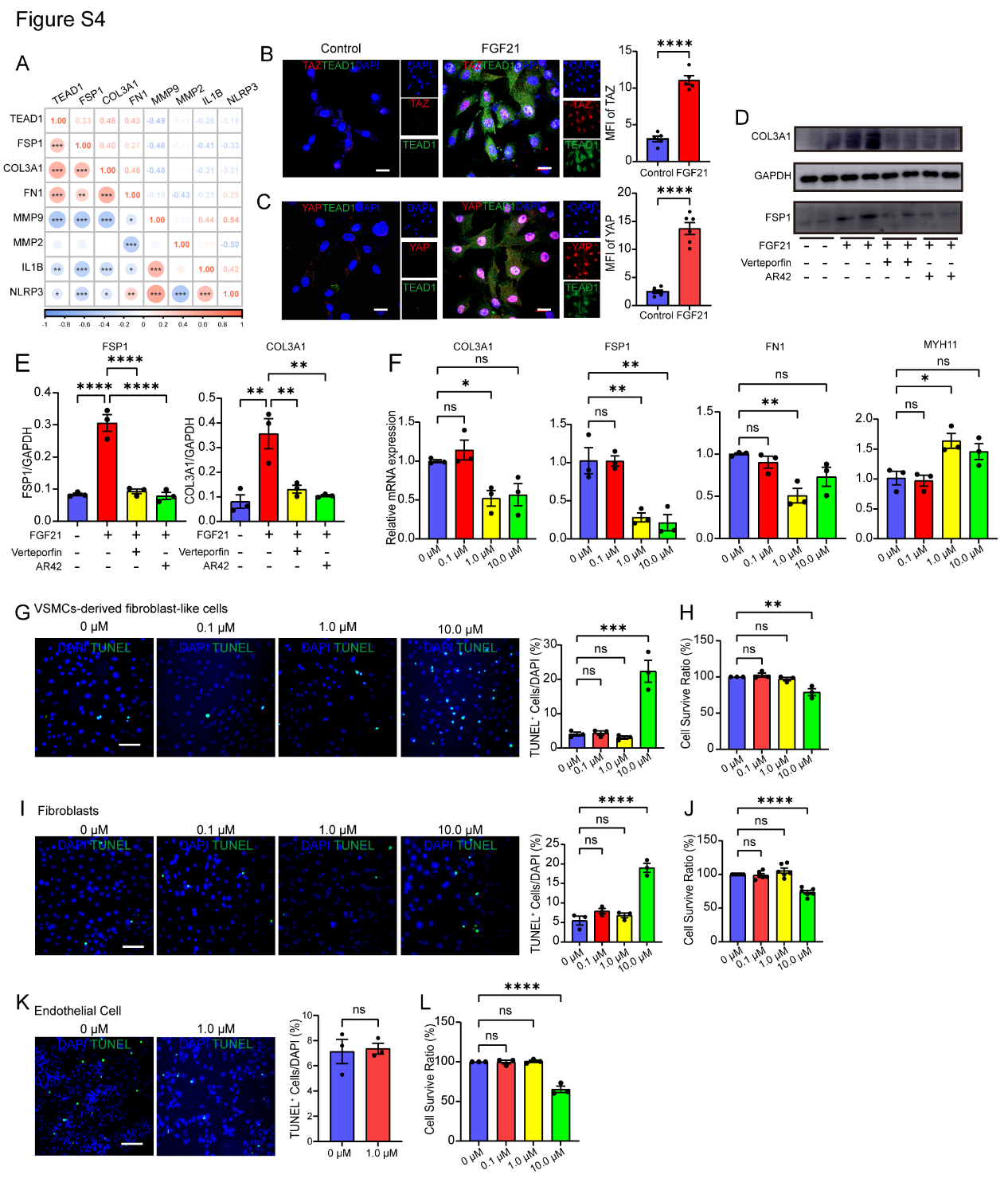


**Figure S4. The function of TEAD1 was dependent on its co-activators YAP or TAZ. A**. The correlation analysis among TEAD1 and fibroblast markers or inflammatory markers in human atherosclerotic plaque dataset (GSE21545). **B**. The representative images and quantification of TAZ in RASMCs and FGF21-treated RASMCs. Scale bar =20μm. **C**. The representative images and quantification of YAP in RASMCs and FGF21-treated RASMCs. Scale bar =20μm. **D**. The representative WB images of COL3A1 and FSP1 in RASMCs treated with PBS, FGF21, FGF21+TAZ inhibitor (AR42), FGF21+YAP inhibitor (Verteporfin). **E**. Quantification of WB results in **D** (n=3 per group). **F**. The mRNA relative expression levels of COL3A1, FN1, FSP1 and MYH11 in FGF21-stimulated RASMCs treated with different concentrations of VT103 (n=3 per group). **G**. The apoptosis of VSMCs-derived fibroblast-like cells stimulated with different concentrations of VT-103 by TUNEL assay (n=3 per group). Scale bar =50μm. **H**. Cell viability of VSMCs-derived fibroblast-like cells stimulated with different concentrations of VT-103 by CCK8 assay (n=3 per group). **I**. The apoptosis of fibroblasts stimulated with different concentrations of VT-103 by TUNEL assay (n=3 per group). Scale bar =50μm. **J**. Cell viability of fibroblasts stimulated with different concentrations of VT-103 by CCK8 assay (n=3 per group). **K**. The apoptosis of endothelial cells stimulated with different concentrations of VT-103 by TUNEL assay (n=3 per group). Scale bar =50μm. **L**. Cell viability of endothelial cells stimulated with different concentrations of VT-103 by CCK8 assay (n=3 per group). For all panels, error bars represented SE. *P*-value was determined by unpaired two-tailed Student’s *t*-test (**B, C and K**) or one-way ANOVA with Bonferroni post-test (**E, F, G, H, I, J and L**). ns no significance, * *P* < 0.05, ** *P* < 0.01, *** *P* < 0.001, **** *P* < 0.0001.


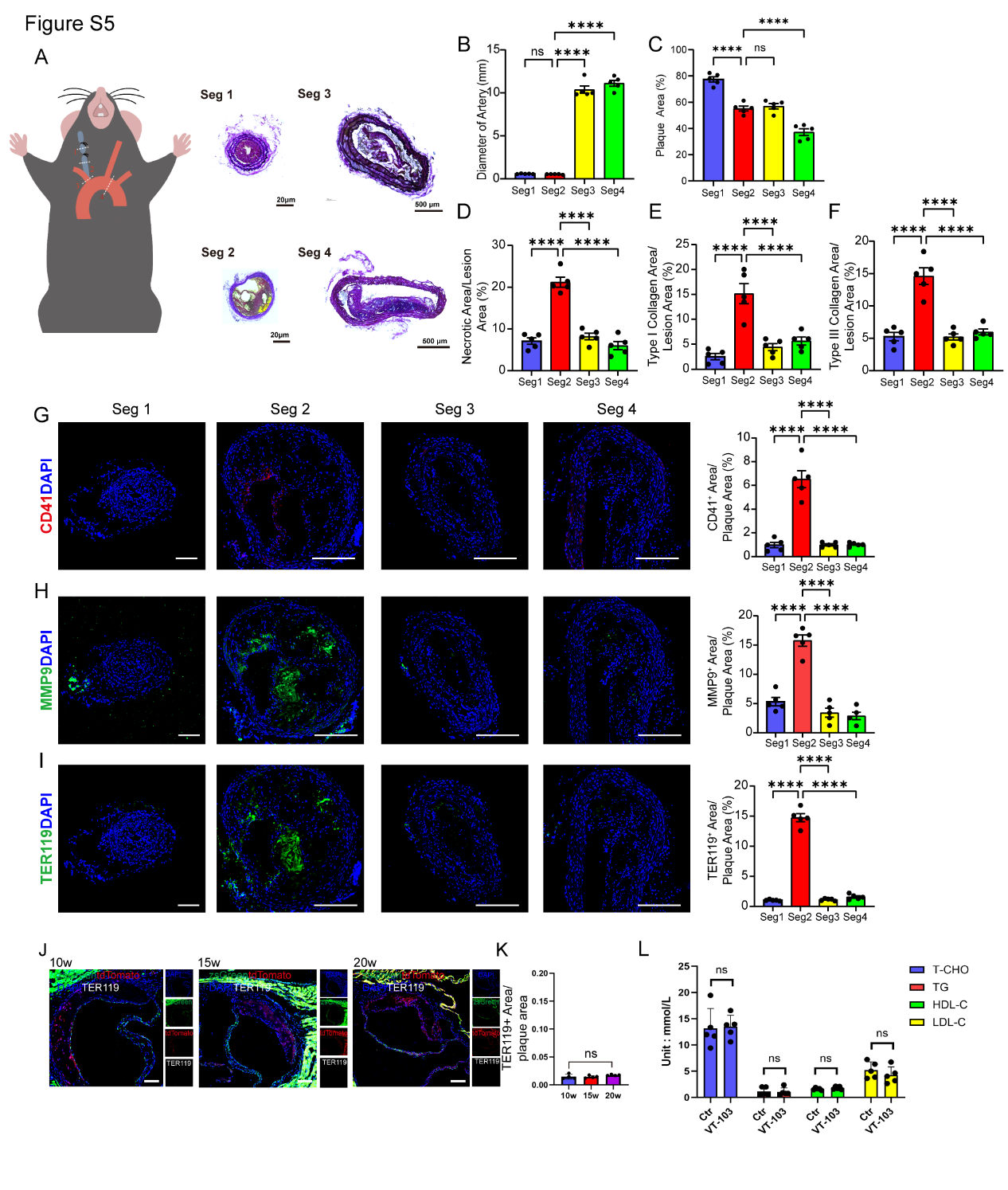


**Figure S5. Assessment of the stability and rupture of different segments in atherosclerotic mice underwent TS surgery. A**. The carton illustrated the different segments in atherosclerotic mice underwent TS surgery and representative MOVAT pentachrome staining of related segments. Scale bar=20μm, 20μm, 500μm and 500μm in seg 1, 2, 3, 4 respectively. **B**. Quantification of artery diameter among different segments in **A** (n=5 per group). **C, D, E and F**. Quantification the percentages of plaque area %, necrotic area/plaque area, type I collagen area/plaque area and type III collagen area/plaque area among different segments in **A** (n=5 per group). **G, H and I**. The representative images and quantification of CD41, MMP9, and TER119 on plaques from different segments (n=5 per group). Scale bar=20μm, 20μm, 500μm and 500μm in seg 1, 2, 3, 4 respectively. **J**. IF staining of TER119 on plaques harvested from mice fed an HFD for 10, 15, and 20 weeks. Scale bar=100μm. **K**. Quantification of the percentage of TER119^+^area/plaque area in **J** (n=4 per group). **L**. The lipid profiles in mice treated with control or VT-103 (n=5 per group). For all panels, error bars represented SE. *P*-value was determined by unpaired two-tailed Student’s *t*-test (**L**) or one-way ANOVA with Bonferroni post-test (**B-K**). ns no significance, * *P* < 0.05, ** *P* < 0.01, *** *P* < 0.001, **** *P* < 0.0001.

**
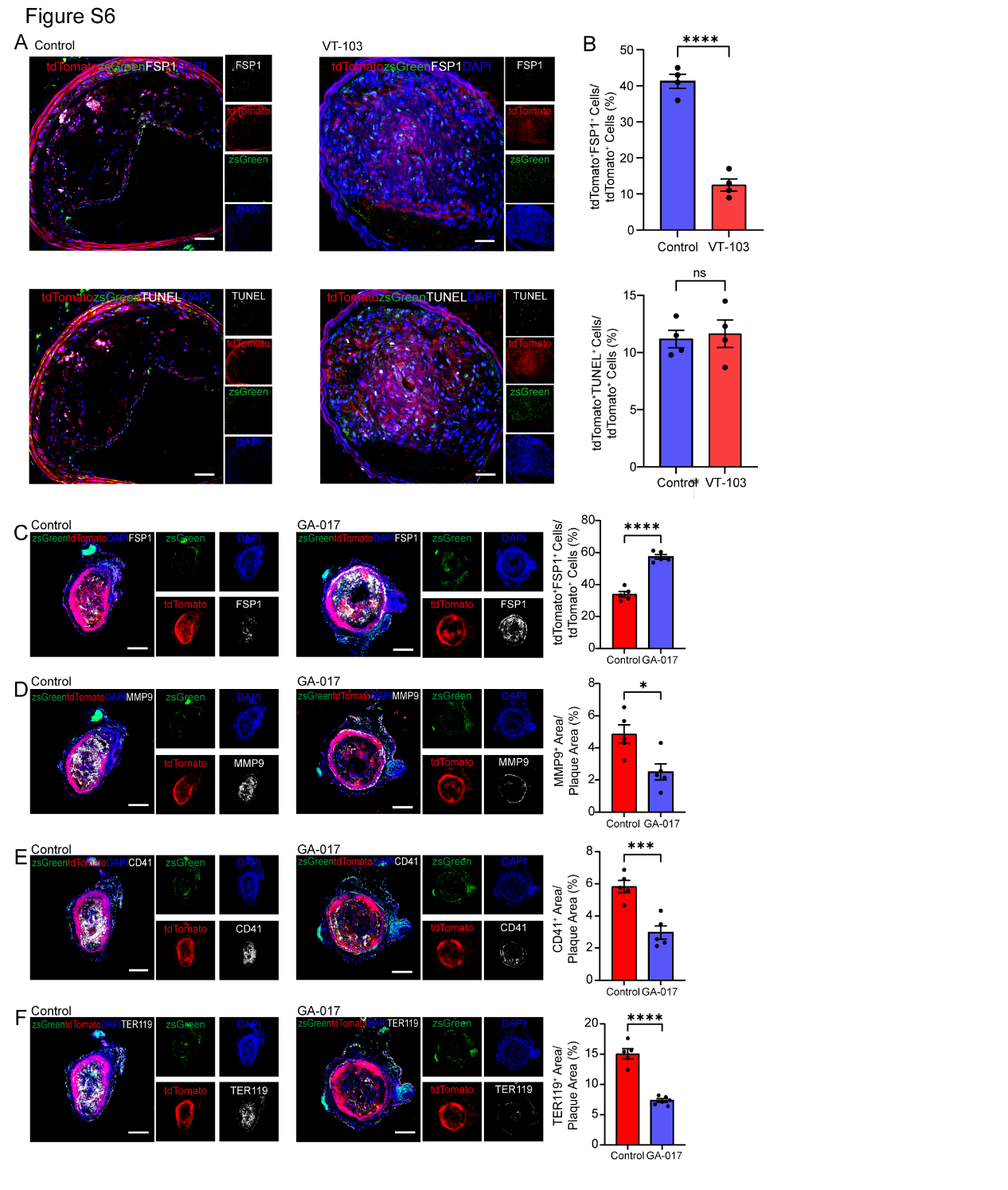
**

**Figure S6. YAP/TAZ enhancer (GA-017) promoted the stability and healing after plaque rupture. A**. IF staining of TUNEL and FSP1 on the series plaques from *Myh11^Cre^ B6G/R Ldlr^-/-^* mice that underwent TS surgery treated with either control or VT-103. Scale bar=100μm. **B**. Quantification the percentage of tdTomato^+^FSP1^+^ cells/tdTomato^+^ cells, and tdTomato^+^TUNEL^+^ cells/tdTomato^+^ cells in **A** (n=4 per group). **C, D, E and F**. The representative images and quantification of IF staining of FSP1, CD41, MMP9, and TER119 on plaques from mice underwent TS surgery treated with control or GA-017 (n=5 per group). Scale bar = 100μm. For all panels, error bars represented SE. *P*-value was determined by unpaired two-tailed Student’s *t*-test (**B-F**). ns no significance, * *P* < 0.05, ** *P* < 0.01, *** *P* < 0.001, **** *P* < 0.0001.


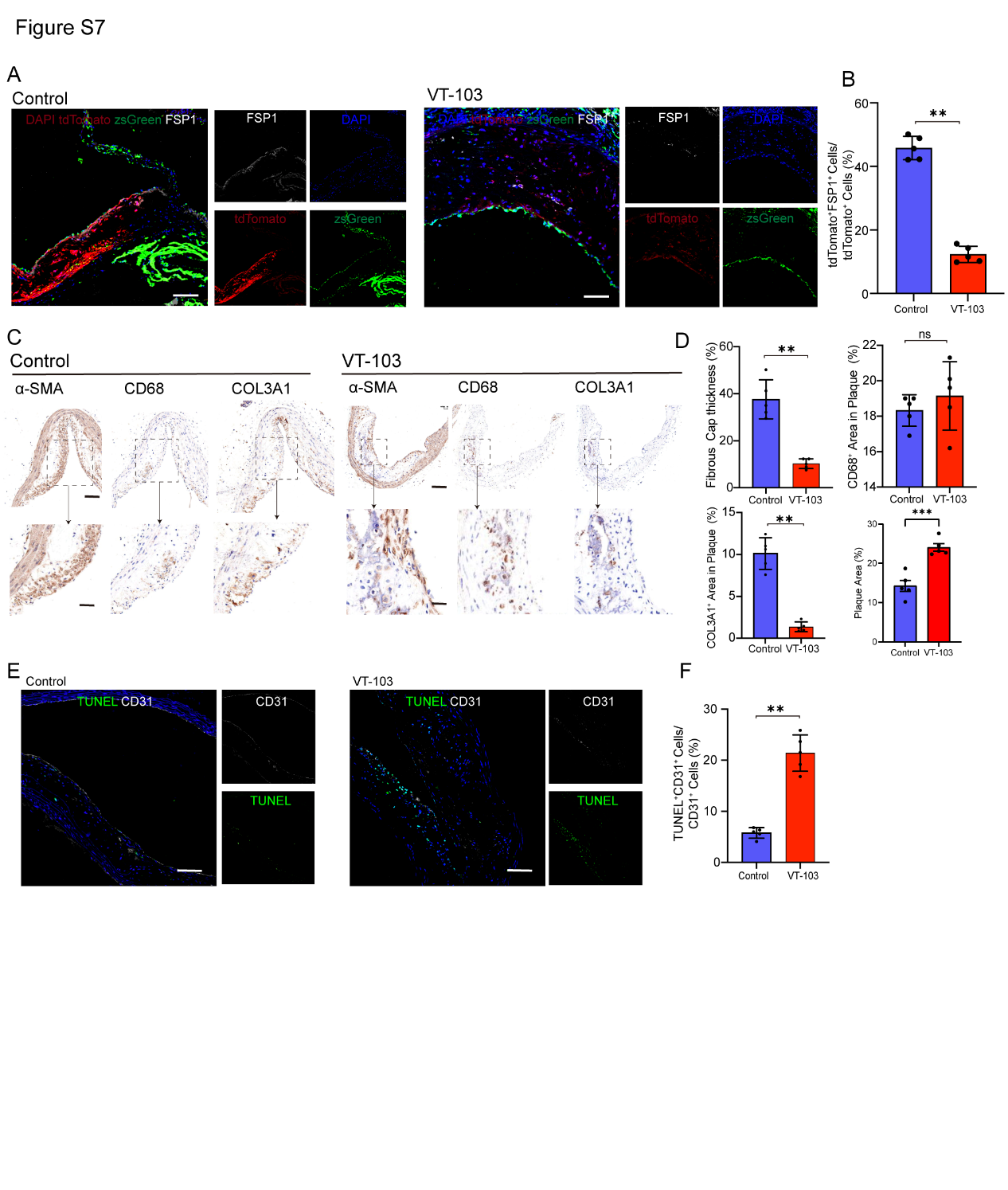


**Figure S7. TEAD1 inhibitor VT-103 attenuated plaque stability and reendothelialization. A**. IF staining of FSP1 on plaques from HFD-fed *Ldlr^-/-^* mice treated with control or VT-103. Scale bar=100μm **B**. Quantification of the percentage of tdTomato^+^FSP1^+^ cells/tdTomato^+^ cells in **A** (n=5 per group). **C**. Representative images of α-SMA, CD68 and COL3A1 on plaques from HFD-fed *Ldlr^-/-^* mice treated with control or VT-103. Scale bar=100μm and 50μm respectively. **D**. Quantification of **C** (n=5 per group). **E**. IF co-staining of TUNEL and CD31 on the plaques from HFD-fed *Ldlr^-/-^* mice treated with control or VT-103.Scale bar=100μm. **F**. Quantification of the percentage of TUNEL^+^CD31^+^ cells/CD31^+^ cells in **E** (n=5 per group). For all panels, error bars represented SE. *P*-value was determined by unpaired two-tailed Student’s *t*-test (**B, D and F**). ns no significance, * *P* < 0.05, ** *P* < 0.01, *** *P* < 0.001, **** *P* < 0.0001.


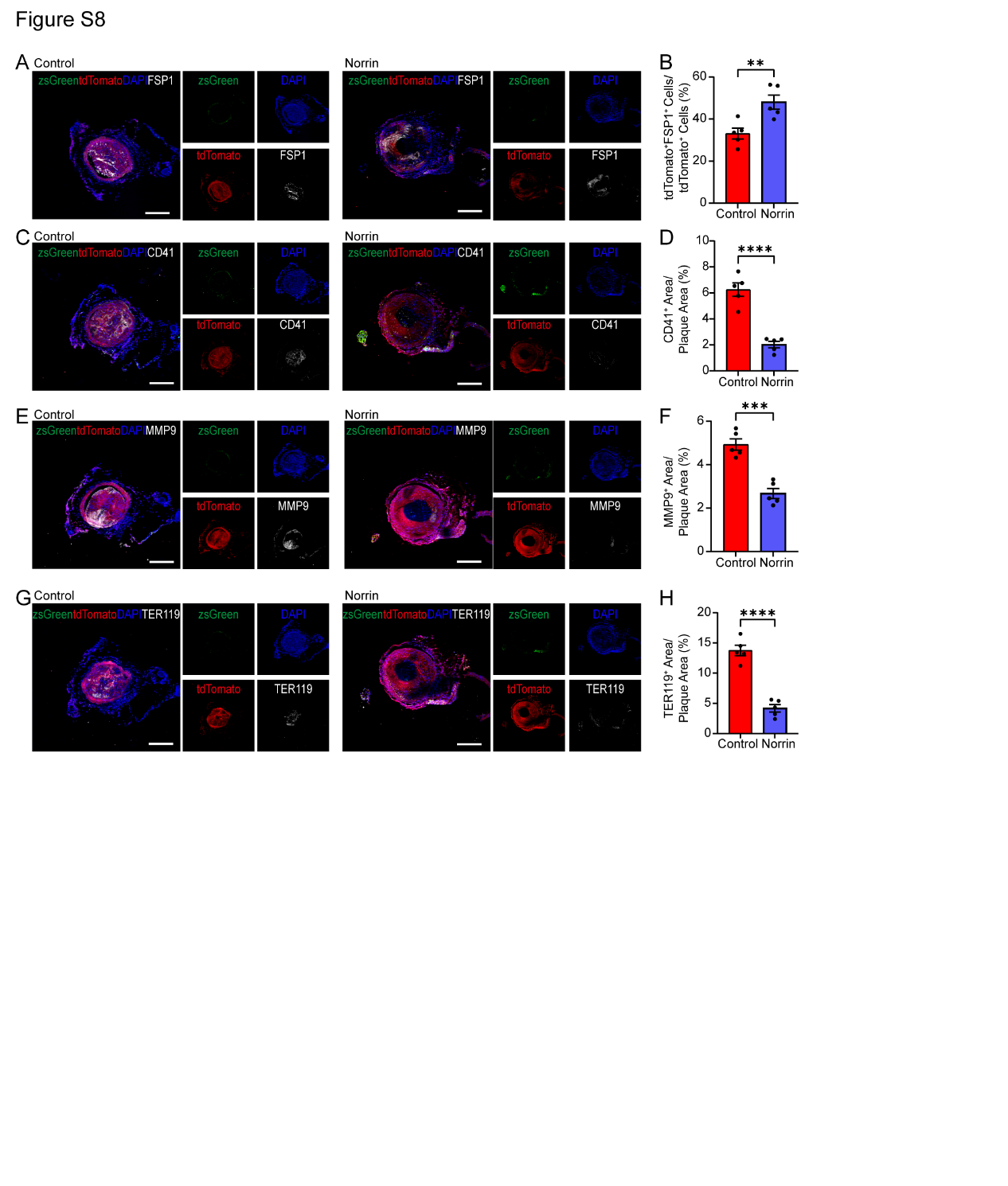


**Figure S8**. **The Wnt4/β-Catenin enhancer Norrin promoted the stability and healing after plaque rupture. A and B**. The representative images and quantification of IF staining of FSP1 on plaques from mice underwent TS surgery treated with control or Norrin (n=5 per group). Scale bar =100μm. **C and D**. The representative images and quantification of IF staining of CD41 on plaques from mice underwent TS surgery treated with control or Norrin (n=5 per group). Scale bar=100μm. **E and F**. The representative images and quantification of IF staining of MMP9 on plaques from mice underwent TS surgery treated with control or Norrin (n=5 per group). Scale bar=100μm. **G and H**. The representative images and quantification of IF staining of TER119 on plaques from mice underwent TS surgery treated with control or Norrin (n=5 per group). Scale bar=100μm. For all panels, error bars represented SE. *P*-value was determined by unpaired two-tailed Student’s *t*-test (**B, D, F and H**). ns no significance, * *P* < 0.05, ** *P* < 0.01, *** *P* < 0.001, **** *P* < 0.0001.


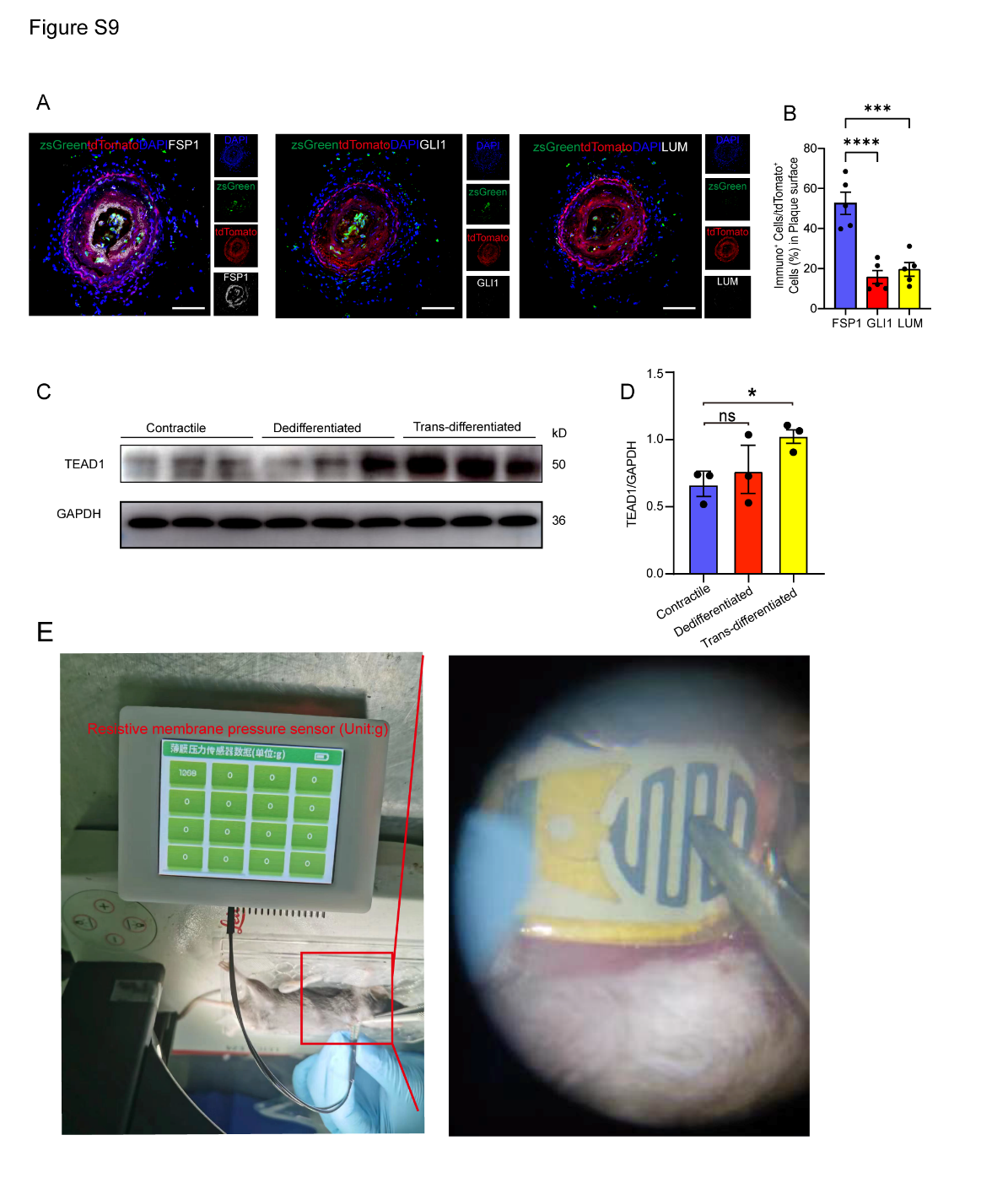


**Figure S9.** **The distribution of tdTomato^+^ FSP1^+^ cells, tdTomato^+^ GLI1^+^ cells, and tdTomato^+^ LUM^+^ cells after plaque rupture. The expression of TEAD1 in three VSMCs phenotypes. Measurement of the forces applied by hemostatic forceps on** **brachiocephalic artery. A.** IF staining of FSP1, GLI1 and LUM on plaques form mice underwent TS surgery. Scale bar=100μm. **B**. Quantification the percentage of tdTomato^+^FSP1^+^ cells/tdTomato^+^ cells, tdTomato^+^GLI1^+^ cells/tdTomato^+^ cells, and tdTomato^+^LUM^+^ cells/tdTomato^+^ cells in **A** (n=5 per group). **C.** The representative WB of TEAD1 among VSMCs, de-differentiated VSMCs, and VSMCs-derived fibroblast-like cells. **D**. Quantification of WB results in **C**. **E**. The image on the left displayed measurement obtained using a membrane pressure sensor, and the zoomed-in image on the right illustrated the measurement area near the brachiocephalic artery in vivo. For all panels, error bars represented SE. *P*-value was determined by one-way ANOVA with Bonferroni post-test (**B and D**). ns no significance, * *P* < 0.05, ** *P* < 0.01, *** *P* < 0.001, **** *P* < 0.0001.
